# PRAT Proteins Operate in Organellar Protein Import and Export in *Arabidopsis thaliana*

**DOI:** 10.3390/plants10050958

**Published:** 2021-05-11

**Authors:** Claudia Rossig, John Gray, Oscar Valdes, Armin Springer, Sachin Rustgi, Diter von Wettstein, Christiane Reinbothe, Joachim Rassow, Steffen Reinbothe

**Affiliations:** 1 Laboratoire de Génétique Moléculaire des Plantes, Université Grenoble-Alpes, BP53F, 38041 Grenoble, France; claudia.rossig@gmx.de (C.R.); oscar.valdes@myubt.de (O.V.); bt434375@myubt.de (C.R.); 2 Department of Biological Sciences, University of Toledo, 2801 West Bancroft Street, Toledo, OH 43606, USA; jgray5@utnet.utoledo.edu; 3 Medical Biology and Electron Microscopy Centre, University Medical Center Rostock, Strempelstraße 14, 18057 Rostock, Germany; armin.springer@med.uni-rostock.de; 4 Department of Plant and Environmental Sciences, Pee Dee Research and Education Center, Clemson University, Florence, SC 29506, USA; srustgi@clemson.edu; 5 Department of Crop and Soil Sciences, Washington State University, Pullman, WA 99164, USA; diter@wsu.edu; 6 Department of Cell Biochemistry, Institute for Biochemistry and Pathobiochemistry, Ruhr-University Bochum, Universitätsstraße 150, 44780 Bochum, Germany; joachim.rassow@ruhr-uni-bochum.de

**Keywords:** chloroplast and mitochondrial membrane transport, protein translocation, preprotein and amino acid transporter (PRAT) family, plant greening and senescence

## Abstract

Chloroplasts need to import preproteins and amino acids from the cytosol during their light-induced differentiation. Similarly, chloroplasts have to export organic matter including proteins and amino acids during leaf senescence. Members of the PRAT (preprotein and amino acid transporter) family are candidate transporters for both processes. Here, we defined the role of two small PRAT gene families, At4g26670 and At5g55510 (HP20 subfamily) versus At3g49560 and At5g24650 (HP30 subfamily) during greening of etiolated plants and during leaf senescence. Using a combination of reverse genetics, protein biochemistry and physiological tools, evidence was obtained for a role of chloroplast HP20, HP30 and HP30-2 in protein, but not amino acid, import into chloroplasts. HP20, HP30 and HP30-2 form larger complexes involved in the uptake of transit sequence-less cytosolic precursors. In addition, we identified a fraction of HP30-2 in mitochondria where it served a similar function as found for chloroplasts and operated in the uptake of transit sequence-less cytosolic precursor proteins. By contrast, HP22 was found to act in the export of proteins from chloroplasts during leaf senescence, and thus its role is entirely different from that of its orthologue, HP20. HP22 is part of a unique protein complex in the envelope of senescing chloroplasts that comprises at least 11 proteins and contains with HP65b (At5g55220) a protein that is related to the bacterial trigger factor chaperone. An ortholog of HP65b exists in the cyanobacterium *Synechocystis* and has previously been implicated in protein secretion. Whereas plants depleted of either HP22 or HP65b or even both were increasingly delayed in leaf senescence and retained much longer stromal chloroplast constituents than wild-type plants, HP22 overexpressors showed premature leaf senescence that was associated with accelerated losses of stromal chloroplast proteins. Together, our results identify the PRAT protein family as a unique system for importing and exporting proteins from chloroplasts.

## 1. Introduction

Plastids are hall-mark organelles of plants [1,2,3,4]. They comprise a family of partially inter-convertible forms, all originating from a simple progenitor called proplastid [1,2]. When seedlings develop in the dark and thus undergo skotomorphogenesis, proplastids differentiate into etioplasts. Upon illumination, etioplasts develop further into chloroplasts. This developmental step, called photomorphogenesis, is associated with the establishment of the photosynthetic apparatus and involves the synthesis of nucleus-encoded and plastid-encoded photosynthetic proteins [2,5,6]. Photosynthetic proteins encoded in the nucleus are synthesized as precursors with NH_2_-terminal transit sequences referred to as transit peptides and must be imported post-translationally from the cytosol. Similarly, amino acids for the biosynthesis of plastid-encoded proteins must be imported from the cytosol.

Components have been identified that mediate the uptake of nucleus-encoded cytosolic precursor proteins into chloroplasts. These form translocon complexes in the outer and inner plastid envelope membranes named the TOC and TIC machineries [7,8,9,10,11]. Current evidence suggests that photosynthetic precursors bind to a surface-exposed receptor dubbed TOC159 and also interact with a second receptor named TOC33. The latter is supposed to transfer the precursor into the import channel formed by another TOC protein named TOC75. Once the precursors have passed this channel they interact with a set of TIC proteins of which TIC110 is supposed to be the actual translocation pore for import across the inner plastid envelope membrane [12].

The import system for amino acids needed for the biosynthesis of plastid-encoded proteins is less well characterized. Candidate transporters that may mediate this uptake step have been identified by proteomics, in silico, and other approaches [13,14,15,16]. An interesting group of plastid envelope proteins that could be involved in amino acid transport are the PRAT proteins comprising preprotein and amino acid transporters of prokaryotic origin [17,18,19]. The PRAT (preprotein and amino acid transporter) family in *Arabidopsis thaliana* comprises 17 members forming 6 different phylogenetic clades [17,18,19]. The PRAT family is composed of At1g72750, At1g17530 and At3g04800 (TIM23 [translocon of the inner mitochondrial membrane protein of 23 kDa] subfamily), At2g37410, At1g20350 and At5g11690 (TIM17 subfamily), At1g18320 and At3g10110 (TIM22 subfamily), At4g16160, At2g28900 and At3g62880 (OEP16-like subfamily), At4g26670 and At5g55510 (HP20 subfamily), as well as At3g49560 and At5g24650 (HP30 subfamily) [17,18,19]. Another protein with similarity in the PRAT motif is the amino acid permease LivH of *Escherichia coli* [20] that together with its homologues in prokaryotes forms a separate phylogenetic clade [17]. Based on the topology of the four founding members of the PRAT family in eukaryotes, TIM17, TIM22, TIM23 and the 16 kDa outer plastid envelope protein OEP16, PRAT proteins were proposed to form channels comprising four hydrophobic trans-membrane α-helices and 3 hydrophilic loops [17]. The characteristic PRAT motif is located in the central region forming the second and third trans-membrane helices [17]. Deviations from this general pattern of trans-membrane domain and PRAT motif organization have been reported [19].

Many of the PRAT proteins identified thus far obviously operate in protein translocation into mitochondria [21,22]. Yeast TIM23, TIM22 and TIM17, for example, establish two distinct translocases in mitochondria that are responsible for the import of cytosolic precursor proteins into and across the inner mitochondrial membrane [21,22]. TIM22 is involved in the import of carrier proteins and other, hydrophobic membrane proteins lacking cleavable NH_2_-terminal presequences [21,22]. By contrast, TIM17 and TIM23 form the second inner mitochondrial membrane translocase that is involved in the uptake of cytosolic proteins containing cleavable NH_2_-terminal presequences [23,24]. Hereby, TIM23′s function is that of a presequence receptor and voltage-gated channel, whereas TIM17 was proposed to accomplish a role as voltage sensor [23,24]. Although the core components of the TIM17:23 and TIM22 translocases are conserved in yeast, animals and plants, differences have been reported for auxiliary components [25,26].

Although most of the mitochondrial PRAT protein family members in plants could accomplish similar roles as their yeast and animal counterparts, other functions are possible. It was proposed that some of the previously identified hypothetical proteins (HPs) in the chloroplast envelope, such as HP30-2 and HP30, are involved in tRNA import into plant mitochondria [27,28]. Other HP family members could operate in amino acid transport, which is not only required during photomorphogenesis but also during leaf senescence. The latter is an active process that involves massive changes occurring at the morphological and gene expression levels [29,30,31,32,33]. Hallmark events include the reduction in the number and size of chloroplasts as well as the disassembly of the photosynthetic apparatus in the thylakoids [34,35,36]. Mass degradation of both stroma and thylakoid membrane proteins has been reported and could lead to a need to export the liberated amino acids from senescing chloroplasts to the cytosol and other destinations.

In the present work, the roles of the two least characterized PRAT gene families were characterized. These include the HP20 subfamily, comprising At4g26670 and At5g55510, and the HP30 subfamily, comprising At3g49560 and At5g24650 [19]. At4g26670 (HP20) and At5g55510 (HP22) encode proteins with 79% amino acid sequence identity, whereas At3g49560 (HP30) and At5g24650 (HP30-2) encode proteins sharing 83% amino acid sequence identity [19]. Here, we examined the roles of all four PRAT proteins both during photomorphogenesis and leaf senescence and found novel results. Using a reverse genetic approach, in combination with biochemical and physiological tests, we report on HP20, HP30 and HP30-2 as being involved in protein, but not amino acid, import into chloroplasts, and on HP22 as being involved in a, thus far unrecognized, protein export pathway from chloroplasts that specifically operates during leaf senescence in *A. thaliana*.

## 2. Results

### 2.1. HP20 and HP30 Interact during the Import of Transit Sequence-Less Precursors into Chloroplasts

**Figure 1:**
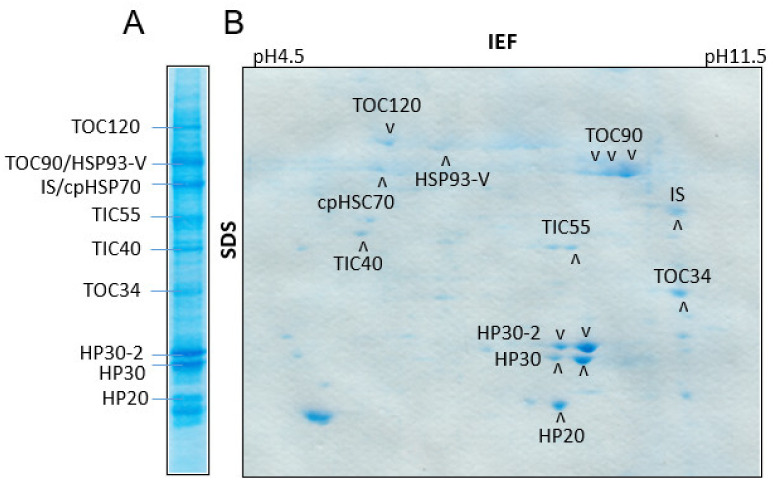
Identification of HP20 and HP30 as components operating in the import of transit sequence-less proteins into chloroplasts. (**A**) One-dimensional pattern of import intermediate-associated proteins (IAPs) formed with ^35^S-ceQORH-(His)_6_. (**B**) as (**A**), but showing a two-dimensional separation comprising isoelectric focusing (IEF) in the first dimension (from left to right) and SDS-PAGE gradient gel electrophoresis (SDS), from top to bottom, in the second dimension. Proteins were stained with Coomassie.

HP20, HP30 and HP30-2 were previously reported to function in the uptake of transit sequence-less cytosolic precursors into chloroplasts [37,38,39]. Using the chloroplast envelope quinone oxidoreductase homologue ceQORH as model for transit sequence-less cytosolic precursors [40], we asked whether HP22 is involved in this new pathway of protein import, too. Plastid envelope import intermediates were produced with hexa-histidine ([His]6)-tagged, 35S-methionine-labeled ceQORH (ceQORH-[His]6) that had been expressed in *E. coli* and purified [37,38,39]. Incubations were carried out with isolated Arabidopsis chloroplasts at 0.1 mM Mg-GTP and 0.1 mM Mg-ATP, nucleoside triphosphate concentrations that favor protein translocation across the outer envelope membrane and also permit interactions of importing proteins with components of the inner plastid envelope import machinery [37,39]. After 15 min, the plastids were re-isolated on Percoll and ruptured under hypertonic conditions. Mixed outer (OM) and inner (IM) envelope membranes were isolated from ruptured chloroplasts and further separated by centrifugation on 20–38% sucrose gradients [41]. Import intermediate-associated proteins (IAPs) were detergent-solubilized from OM-IM junction complexes established by ceQORH-(His)6 as described in [41]. After purification by Ni-NTA chromatography [37,39] and 2D SDS-PAGE, IAPs were stained with Coomassie and identified by manual or automated protein sequencing. Figure 1 shows that the isolated IAPs comprised at least 10 different proteins. Among these IAPs were HP20, HP30 and HP30-2. Other IAPs comprised TOC120, TOC90 and TOC34, representing members of the TOC superfamily of presequence receptors [42,43,44], HSP93-V and cpHSC70, representing members of the heat shock protein and heat shock cognate protein families [45,46], TIC40 that is presumed to act as docking protein for HSP93-V, as well as TIC55 being involved in redox control (Table S1). Their interactions confirmed that chloroplasts make use of a unique combination of PRAT proteins, TOC components as well as molecular chaperones for importing transit sequence-less precursor proteins [37,38,39].

### 2.2. Isolation and Characterization of HP20 and HP30 Knock-Out Mutants of A. thaliana

T-DNA insertion lines were obtained from the SALK Institute [47] and screened for homozygous knock-out mutants for HP20 and HP30. Individual plants of the T3 generation were tested for homozygosity by PCR [48] and the position of the T-DNA insertion was mapped by sequencing PCR products obtained with a combination of forward and reverse primers specific for the gene of interest and the left border of the T-DNA (Figures S1 and S2, panels A and F).

Sequence analysis revealed the same position of the T-DNA insertions in lines Athp20;2 and Athp20;3 as well as in lines Athp30;1 and Athp30;2, respectively. Therefore, only one of the two mutants each was further analyzed. A second, independent mutant allele for each gene was identified by Athp20;1 and Athp30;3, respectively (Figures S1 and S2). For the four selected knock-out lines, designated Athp20;1, Athp20;2, Athp30;2 and Athp30;3, homozygous plants were obtained and characterized by Southern, Northern and Western blotting, as well as growth tests on Murashige-Skoog (MS)-agar medium containing kanamycin. The relevant data are presented in Figures S1 and S2.

Phenotypic characterization of the Athp20;1, Athp20;2, Athp30;2 and Athp30;3 mutants revealed no differences in plant habit (Figure S3A) as well as similar plastid numbers and ultra-structures as compared with wild-type plants (Figure S3B). Moreover, the synthesis and accumulation patterns of total leaf and plastid proteins in light-grown Athp20;1, Athp20;2, Athp30;2 and Athp30;3 plants were indistinguishable from those of 4-weeks-old, adult wild-type plants (Figure S3C,D; see also Figure S4A,B), indicating that HP20 and HP30 are dispensable for bulk amino acid uptake into chloroplasts in adult plants. If such uptake defect would occur, Athp20;1, Athp20;2, Athp30;2 and Athp30;3 chloroplasts should have lowered rates of 35S-methionine-driven plastid protein synthesis which was not the case.

Greening experiments under low white light conditions (30–40 µE m^−2^ s^−1^) suggested some delay in chlorophyll accumulation to occur for etiolated Athp20;1 and Athp20;2 seedlings during the first 6–8 h of illumination, as compared to wild-type and Athp30;2 and Athp30;3 seedlings (Figure S5 and Table S2). This correlated with reduced levels of the reaction center protein D1 of photosystem II, the α-subunit of cytochrome b-559, the 33 kDa subunit of the oxygen evolving complex, OEC33, and the ribulose-1,5-bisphosphate carboxylase/ oxygenase (RubisCO) large and small subunits (Figures S6 and S7). Interestingly, a ca. 4–5-fold up-regulation of the early light-inducible protein 1 (ELIP1) was observed in Athp20 mutant seedlings (Figures S6 and S7). All these effects were overcome at later stages of development (cf. Figure S5 vs. Figure S3 and Table S2). Greening experiments under high light conditions (≈210 µE m^−2^ s^−1^) followed by seedling viability tests using the tetrazolium dye demonstrated that Athp20 and Athp30 seedlings were fully viable and did not photo-bleach as did the Atoep16-1;1 mutant used as reference [49] (Figure S8A,B). Pulse-labeling of leaf proteins with 35S-methionine showed unaltered patterns of nucleus-encoded and plastid-encoded plastid proteins during greening of Athp20;1, Athp20;2, Athp30;2 and Athp30;3 seedlings (Figure S8C,D). In amino acid uptake assays with 35S-methionine, no difference became apparent for plastids from etiolated and greening Athp20, Athp30 and wild-type plants (Figure S9 and Table S2). On the basis of all of these findings we concluded that HP20 and HP30 do not act as bulk amino acid transporters during greening.

### 2.3. Etiolated Athp30-2 Seedlings Show Reduced Viability during Greening

**Figure 2:**
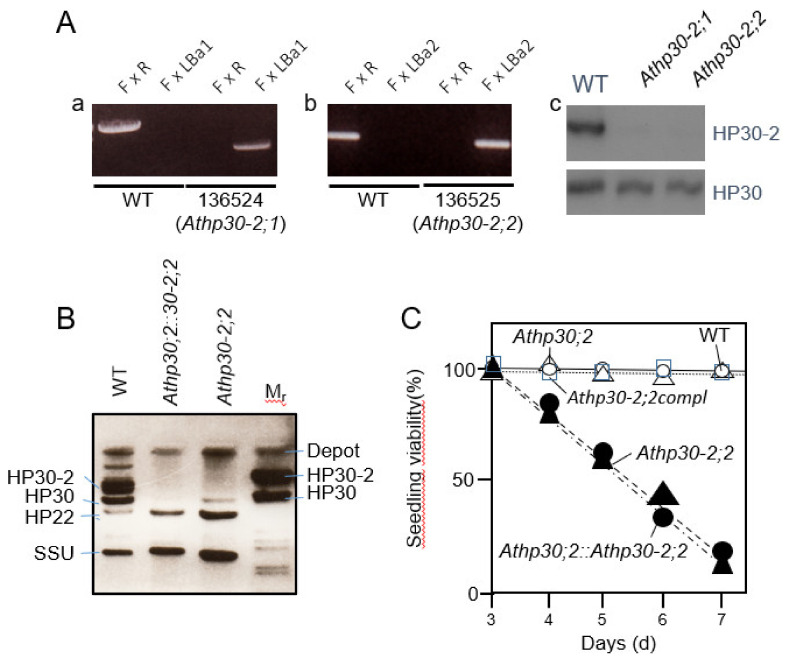
Characterization of *Athp30-2;1* (SALK_ 136524) and *Athp30-2;2* (SALK_ 136525) mutants. (**A**) Identification of Athp30-2;1 (SALK_136524) (**a**) and Athp30-2;2 (SALK_136525) (**b**) through genotyping using the indicated primer combinations and confirmation of the absence of HP30-2 protein in Athp30-2;1 and Athp30-2;2 versus wild-type (WT) plants through Western blotting using a mono-specific antibody (**c**, upper panel). For reference, a replicate protein gel blot was probed with HP30 antibodies (**c**, lower panel). (**B**) Absence of HP30-2 protein in Athp30-2;2 (SALK_136525) single and Athp30-2;2 (SALK_136525)::Athp30;2 (SALK_112126) double mutants versus wild-type (WT) plants. Shown is a Western blot of chloroplast proteins that was simultaneously probed with mono-specific antisera against HP22, HP30 and HP30-2 as well as the small subunit of ribulose-1,5-bisphosphate carboxylase/oxygenase (SSU). (**C**) Seedling viability of Athp30;2 (SALK_112126)(open triangles) and Athp30-2;2 (SALK_136525)(filled triangles) single mutants, and Athp30;2:: Athp30-2;2double mutant plants (dots), as compare to the wild-type (WT)(open circles) and Athp30-2;2 expressing the HP30-2 cDNA (squares).

**Figure 3:**
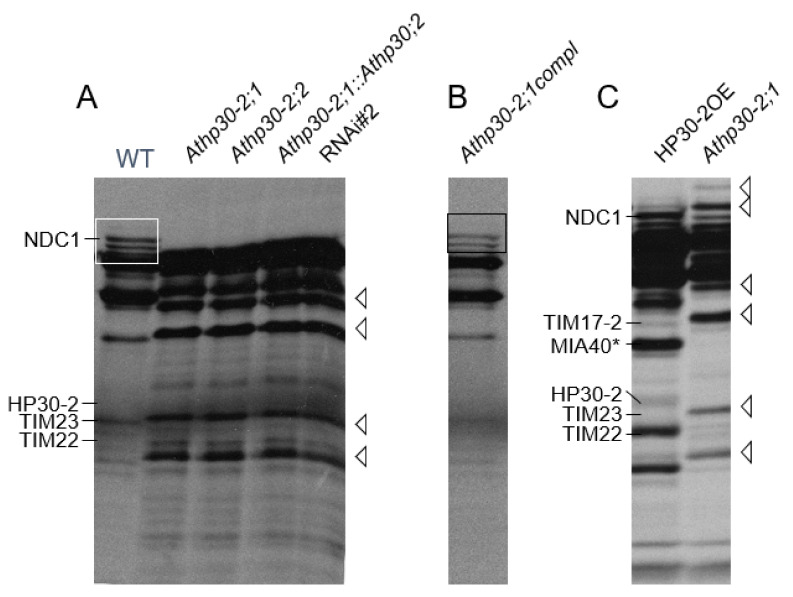
Analysis of inner mitochondrial membrane proteins in *Athp30-2* and *Athp30* mutant plants. (**A**) Pattern of inner mitochondrial membrane proteins in wild-type (WT) plants, *Athp30-2;1* (SALK_136524) and *Athp30-2;2* (SALK_136525) single mutant plants, *Athp30-2;2* (SALK_136525)::*Athp30;2* (SALK_112126) double mutant plants, and *RNAi* plants lacking both HP30 and HP30-2 (*RNAi#2*; cf. Rossig et al., 2013 and 2017). (**B**) Pattern of inner mitochondrial membrane proteins in *Athp30-2;1* (SALK_136524) plants that had been genetically complemented with the HP30-2 cDNA (*Athp30-2;1compl)*. (**C**) Pattern of inner mitochondrial membrane proteins in wild-type plants overexpressing the HP30-2 cDNA, as compared to *Athp30-2;1* (SALK_136524) mutant plants. NDC1 defines the C subunit of the alternative NAD(P)H dehydrogenase, MIA40 defines the 40 kDa mitochondrial intermembrane space import and assembly protein, and TIM17-2, TIM22 and TIM23 define key translocase components operative in the import of cytosolic precursor proteins into and across the inner mitochondrial membrane.

HP30-2 and HP30 are closely related and share 83% amino acid sequence identity [19,28,37]. In previous work, RNA interference (RNAi) lines lacking both HP30-2 and HP30 were shown to be seedling-lethal during greening [28,37]. Because HP30-2 is present both in chloroplasts and mitochondria [19,27,28], it was not possible in our previous work to discern at which place HP30-2 may primarily operate to control seedling viability *in planta*. To answer this question, knock-out mutants were identified for At5g24650 (HP30-2) and characterized further. Genotyping demonstrated the presence of three independent alleles of homozygous *Athp30-2* plants that were designated *Athp30-2;0* (SALK_149871), *Athp30-2;1* (SALK_136524) and *Athp30-2;2* (SALK_136525) (Figure 2A, panels a and b, and data not shown). Expression studies confirmed the absence of HP30-2 protein in *Athp30-2;1* and *Athp30-2;2* seedlings (Figure 2A, panel c, as well as Figure 2B), whereas *Athp30-2;0* (SALK_149871) turned out to be a false-positive mutant still containing HP30-2 protein [27]. Light-grown *Athp30-2;1* and *Athp30-2;2* plants had no visible phenotype if grown under continuous white light illumination but died when grown in darkness and subjected to a white light shift to induce greening (Figure 2C). No such conditional seedling lethality was observed for greening *Athp30;2* plants (Figure 2C). Because etiolated *Athp30-2;2::Athp30;2* double mutant seedlings were similarly light-sensitive as *Athp30-2;2* plants (Figure 2C), we disproved cumulative effects of HP30-2 and HP30 *in planta*. Seedling lethality of *Athp30-2;1* and *Athp30-2;2* plants was overcome by genetically complementing these mutants with the HP30-2 cDNA (Figure 2C). Interestingly, these HP30-2-dependent effects correlated with down-regulation for inner mitochondrial membrane proteins such as TIM22, TIM8, TIM9 and TIM10 in *Athp30-2;1* and *Athp30-2;2*, HP30::HP30-2 *RNAi*, as well as *Athp30;1*::*Athp30-2;2* mutant versus wild-type plants (Figure 3A). At the same time, the amounts of TIM17-2 and TIM23 were up-regulated in *Athp30-2;1* and *Athp30-2;2*, HP30::HP30-2 mutant and *RNAi* plants (Figure 3A,C). As previously reported [28], HP30-2 forms with TIM22, TIM10, TIM9 and TIM8, a translocase for transit sequence-less proteins whose lack may be in part overcome *in planta* by increasing the amounts of TIM17-2 and TIM23. All of the observed differential changes in HP30-2 and TIM protein abundance were abolished by genetically complementing the *Athp30-2;1* mutant with the HP30-2 cDNA (Figure 3B). Overexpression of the HP30-2 cDNA in the wild-type background increased the amounts of HP30-2 and TIM22 as well as MIA40, the 40 kDa mitochondrial intermembrane space import and assembly protein, and NDC1, the type II NAD(P)H quinone oxidoreductase subunit NDC1 (Figure 3C), that have been shown to form distinct complexes with HP30-2 and to operate in normal and oxidative protein folding in the intermembrane space of mitochondria and may additionally be involved in cell death regulation [28].

### 2.4. HP22 Is Part of a Unique Protein Export Pathway from Chloroplasts that Operates during Leaf Senescence

**Figure 4:**
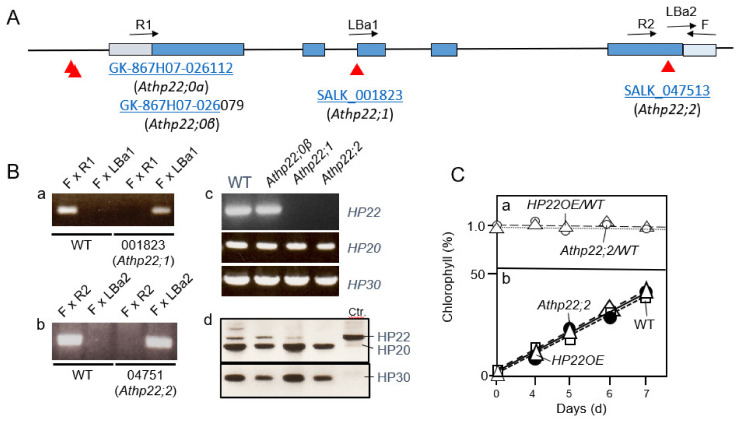
Identification of *Athp22;1* (SALK_001823) and *Athp22;2* (SALK_047513) mutants. (**A**) Structure of the *AtHP22* gene (At5g55510) and positions of T-DNA insertions. (**B**) Genotyping to identify homozygous *Athp22;1* (SALK_001823)(a) and *Athp22;2* (SALK_047513)(b) plants, and demonstration of the absence of HP22 transcript by RT-PCR (c) and HP22 protein by Western blotting using antibodies against HP22 and HP20 (d). Respective PCR and Western blot data for the chosen controls are included (c and d). What does the Ctr in panel B, d stand for? (**C**) Chlorophyll accumulation in 4.5 days-old, dark-grown *Athp22;2* (SALK_047513) mutant and wild-type (WT) seedlings as well as wild-type seedling overexpressing the HP22 cDNA after white light exposure (in hours). The lower panel (b) shows the kinetics of chlorophyll accumulation, whereas the upper panel (a) shows the same data relative to those seen for the wild-type. Because all values are close to 1, there are no differences in the kinetics of chlorophyll accumulation detectable for *Athp22;2* (SALK_047513) mutant (open circles and dots) and HP22-overexpressing plants (open and filled triangles) versus wild-type plants (squares).

The protein isolation data presented in Figure 1 suggested that HP22 is not involved in the import of transit sequence-less precursors into chloroplasts. In order to explore the role of HP22 *in planta*, T-DNA insertion lines were identified (Figure 4A). Of the four potential *Athp22* mutant alleles present in the SALK and Gabi-Kat collections [47,50] (cf. Figure 4A), only two (SALK_001823 and SALK_047513) lacked HP22 transcript (Figure 4B). These were designated *Athp22;1* and *Athp22;2* and characterized further. On protein gel blots carried out on chloroplasts from T_2_ plants, *Athp22;1* (SALK_047513) still contained some HP22 protein, whereas *Athp22;2* (SALK_001823) did not. Thus, mainly *Athp22;2* was used in subsequent experiments and crosses with the *Athp20;1* and *Athp20;1* mutants (cf. Figure 4B vs. Figures S10 and S11).

**Figure 5:**
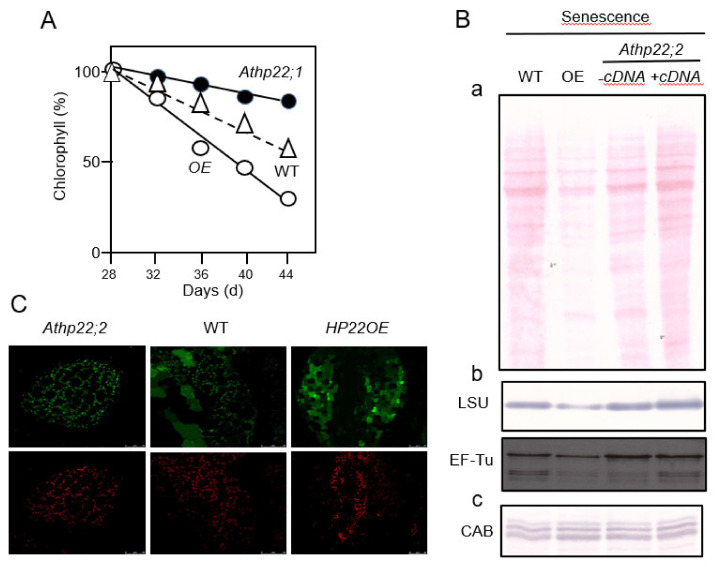
Role of HP22 during leaf senescence. (**A**) Time course of the decay of chlorophyll as a measure of leaf senescence progression in wild-type plants (WT), wild-type plants overexpressing the HP22 cDNA (HP22OE), and *Athp22;2* (SALK_047513) mutant plants. (**B**) Patterns of Poncau S-stained total chloroplast proteins (**a**) and respective Western blot analysis (**b**) on 5 weeks-old wild-type plants (WT), wild-type plants overexpressing the HP22 cDNA (OE), as compared with *Athp22;2* (SALK_047513) mutant plants (− cDNA), and genetically complemented *Athp22;2* (SALK_047513) plants (+ cDNA). Replicate protein gel blots were proved with antibodies (**b** and **c**) against plastid-encoded soluble stromal proteins, such as the large subunit of ribulose-1,5-bisphosphate carboxylase/oxygenase (LSU) and elongation factor EF-Tu (**b**), and nucleus-encoded thylakoid membrane proteins such as the light harvesting proteins of photosystem II (CAB)(**c**). Note that all protein data refer to on an equal plastid number basis. (**C**) Senescence-induced leakage of stromal GFP from chloroplasts *in planta*. Sl-pRC32 (rrn16::accD*)-driven GFP accumulation in chloroplasts was allowed to proceed in leaves of transplastomic *Athp22;2* (SALK_047513), wild-type plants and HP22OE plants. Chlorophyll and GFP fluorescence was collected simultaneously by confocal laser scanning microscopy and compared for the different genotypes. Note the beginning leakage of GFP from chloroplasts in 5 weeks-old wild-type plants that is indicative of senescence progression, the lack of a comparable GFP leakage from chloroplasts in the *Athp22;2* (SALK_047513) mutant, and the acceleration of GFP leakage from chloroplasts in wild-type plants overexpressing HP22. Original scale bars are indicated.

Both the *Athp22;1* and *Athp22;2* mutant had no visible phenotype if grown under 16h light/8 h dark cycles or under continuous white light illumination (data not shown). Moreover, no difference in the kinetics of chlorophyll accumulation during greening was apparent for *Athp22;1* and *Athp22;2* single mutant seedlings, *Athp20::Athp22* double mutant seedlings and wild-type plants overexpressing the HP22 cDNA (Figure 4C and Figure S10). As compared to the wild-type, however, *Athp22;1* and *Athp22;2* mutant plants displayed a significant delay in leaf senescence, reflected by the slower decay in the chlorophyll content (Figure 5A). By contrast, an acceleration in chlorophyll decay indicative of a faster senescence progression was observed for plants overexpressing the HP22 cDNA (Figure 5A). Because *Athp20* and *Athp30* single mutants and *Athp20::Athp30* double knock-out mutants had no phenotypes under conditions of natural as well as abscisic acid (ABA) and jasmonic acid methyl ester (MeJA)-induced, artificial senescence (Figures S11–S17 and Table S4), we concluded a specific and unique role of HP22 *in planta*.

Protein gel blot analyses revealed significantly less total chloroplast protein in 5 weeks-old HP22-overexpressing plants, as compared to wild-type and *Athp22;1* and *Athp22;2* single mutant plants (Figure 5B, panel a). In further studies using antibodies against plastid-encoded stromal proteins, such as the large subunit (LSU) of RuBisCO and the translation elongation factor EF-Tu, some more refined effects were observed (Figure 5B, panel b). The levels of both, LSU and EF-TU, were significantly lower in 5 weeks-old the HP22-overexpressor, as compared to wild-type and *Athp22;1* and *Athp22;2* mutant plants (Figure 5B, panel b). Because parallel assays failed to reveal gross differences in the amounts of the major light-harvesting proteins of photosystem II (CAB gene products) in HP22-overexpressor versus wild-type and *Athp22;1* and *Athp22;2* plants (Figure 5B, panels a and b), we assumed a specific role of HP22 in the export of soluble constituents (stromal proteins) from chloroplasts.

In order to further explore this possibility, transplastomic plants were generated expressing constructs bearing the promoter of the plastid 16S rRNA gene (*rrn16*), one of the strongest promoters in chloroplasts known to date, combined with the 5′–UTR from the *accD* gene encoding a subunit of the acetyl-CoA carboxylase, in front of the green fluorescent protein (GFP) coding region [51]. Confocal laser scanning microscopy on 5 weeks-old plants allowed co-localizing GFP and chlorophyll in chloroplasts of wild-type plants. Co-localization was apparent for most of the leaf areas analyzed, except for some regions where significant amounts of GFP were present in the cytosol. In leaves of *Athp22;2* plants, both fluorescence markers sharply co-localized in chloroplasts in almost all of the leaf samples analyzed (Figure 5C). In marked contrast, there was almost no match between GFP and chlorophyll fluorescence in the leaves of HP22-overexpressing plants (Figure 5C). In every leaf sample analyzed, GFP fluorescence was always spread over large areas of the cytosol, whereas chlorophyll remained chloroplastic (Figure 5C). This result suggested a promotion of chloroplast leakage to occur in HP22-overexpressing plants, causing the rapid release of stromal constituents including plastid-encoded GFP and other markers such as RBCL and EF-Tu in HP22-overexpressing plants. Along with the Western blot data shown before, we concluded that HP22 most likely operated in chloroplast protein exporter in senescing plants.

### 2.5. Isolation of Proteins Interacting with HP22 in Chloroplasts

We next isolated proteins interacting with HP22 in the plastid envelope. HP22 was bacterially expressed as NH_2_-terminally or COOH-terminally hexa-histidine ([His]_6_)-tagged protein, purified and imported into chloroplasts from 5 weeks-old plants, as described in Figure 1. Envelope protein complexes formed with HP22-(His)_6_ in turn were purified from ruptured chloroplasts, separated by SDS-PAGE and individual proteins identified by manual or automated sequencing. Of the 11 main protein bands co-isolated with HP22-(His)_6_ (Figure S18), one prominent band could be identified as being related to a previously characterized acidic, plastid envelope protein of ≈62 (61.733) kDa designated HP65b [52]. HP65b is encoded by At5g55220 in Arabidopsis. It shares amino acid sequence similarity to the bacterial trigger factor chaperone [52,53]. Surprisingly, HP65b contains no predictable trans-membrane domains and is presumed to be synthesized without an NH_2_-terminal chloroplast transit peptide [52]. An orthologue of HP65b is present in *Synechocystis* (Q55511) and was previously suggested to operate in protein secretion from cyanobacteria [52].

**Figure 6:**
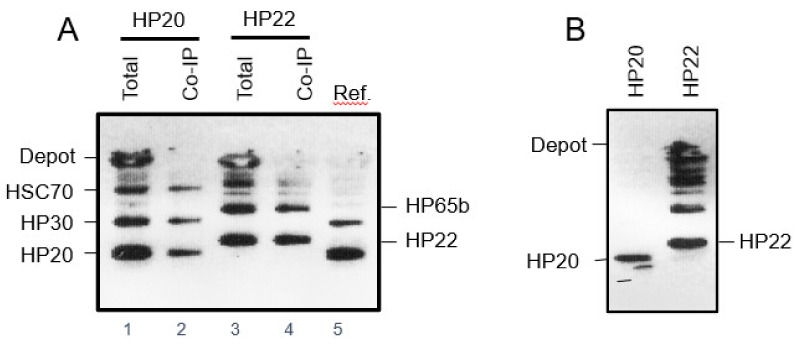
Identification of proteins interacting with HP22 in chloroplasts (**A**) and mitochondria (**B**), respectively. (**A**) Western blot separating plastid envelope proteins interacting with HP20-(His)_6_ and HP22-(His)_6_, respectively (lanes 1 and 3), and analysis of co-immunoprecipitates obtained with antisera against HP20, HP30 and HSC70 for the HP20-(His)_6_-containing complexes (lane 2) and with antibodies against HP22 and HP65b for the HP22-(His)_6_-containing complexes (lanes 3 and 4). Lane 5 depicts the pattern of *in vitro*-expressed HP20 and HP30 used as reference proteins (Ref.). (**B**) Pattern of proteins interacting with HP20-(His)_6_ and HP22-(His)_6_ in isolated Arabidopsis mitochondria. Note the different patterns of HP22-interacting proteins relative to that in chloroplasts (cf. A) and the lack of HP20-interacting proteins in mitochondria.

Antibodies were raised against HP65b. On Western blots, these antibodies recognized a ≈ 60 kDa band (data not shown). In pull-down assays using maltoside-solubilized chloroplast envelope protein extracts, the antibody against HP65b co-precipitated HP22 (Figure 6A), indicating that both proteins interact. By contrast, antibodies against HP20 did not precipitate HP65b but instead precipitated HP30 and stromal HSC70-2 (Figure 6A). Together, these results proved the specificity of the interactions observed here and elsewhere for chloroplasts (cf. Figure 1) [37,39]. When HP22 was imported into isolated Arabidopsis mitochondria, a different pattern of interacting proteins was obtained (Figure 6B). Control studies carried out with HP20 did not reveal any interacting mitochondrial proteins, in agreement with previous proteomics and localization studies demonstrating the absence of HP20 in mitochondria [8,37].

### 2.6. Genetic Interaction of HP22 with HP65b

**Figure 7:**
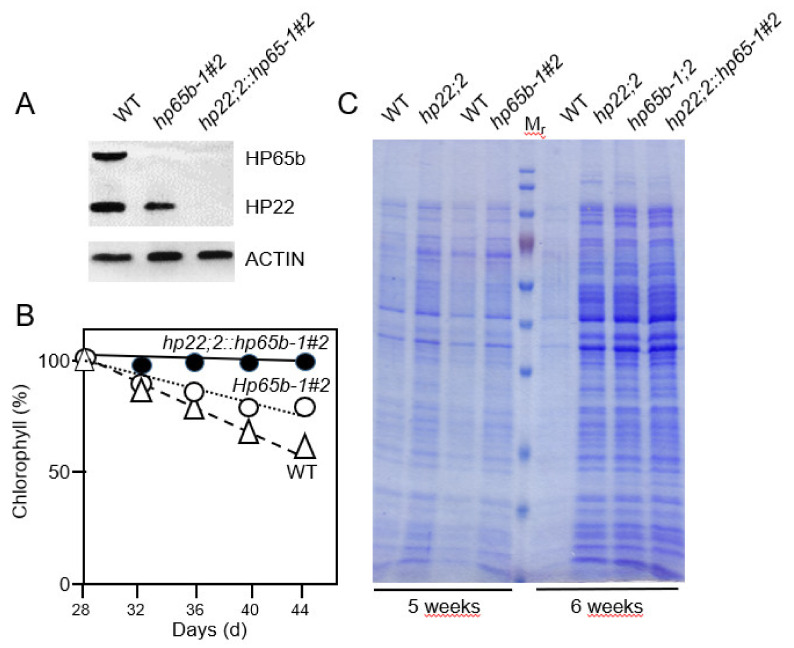
Genetic interaction of HP65b with HP22 during leaf senescence. (**A**) Protein gel blot analysis to demonstrate the lack of HP65b in the generated RNAi line Athp65b-RNAi-1#2 (cf. Figure S19 and S20) and respective cross with Athp22;2 (Athp22;2::Athp65b-RNAi-1#2) versus the wild-type. For comparison, replicate blots were probed with antibodies against HP22 and ACTIN. (**B**) Time course of the decay of chlorophyll in *Athp65b-RNAi-1#2*, *Athp22;2::Athp65b-RNAi-1#2* and wild-type plants undergoing leaf senescence. (**C**) Patterns of Coomassie-stained total chloroplast proteins on an equal plastid number basis in 5- and 6-weeeks-old Arabidopsis plants. Note the severe delay in chloroplast protein loss in *Athp65b--RNAi1#2* and *Athp22;2::Athp65b-RNAi-1#2* versus wild-type plants.

If HP22 and HP65b would interact, their common lack should lead to additive effects and provoke a further retardation of leaf senescence. In order to test this hypothesis, an *RNAi* approach was undertaken to drop the expression of HP65b in the wild-type and *Athp22;2* backgrounds. Of the two sorts of RNAi lines established for wild-type plants, those lacking HP65b transcript and protein were selected and propagated further (Figure S19). Interestingly, all of these lines displayed similar delays in leaf senescence as those found for *Athp22;2* plants. An example was provided by *Athp65b-RNAi-1#2* that was subjected to further in-depth analysis here (Figure 7; see also Figures S19 and S20). When *Athp65b-RNAi-1#2* plants were crossed with the *Athp22;2* mutant, a type of double mutant was obtained in which the expression of both HP65b and HP22 was dropped to undetectable levels (Figure 7A). Significantly, *Athp65b-RNAi-1#2::Athp22;2* plants showed a further delay in senescence progression as compared to their parental genotypes that correlated with a marked retention of stromal chloroplast constituents (Figure 7B,C). We concluded that HP22 and HP65b act synergistically *in planta*.

## 3. Discussion

### 3.1. Function of HP20, HP30 and HP30-2 in Chloroplasts

Chloroplast biogenesis requires the uptake of both nucleus-encoded plastid precursor proteins as well as amino acids from the cytosol. Similarly, chloroplasts export organic matter during leaf senescence. In this study, we asked if two previously characterized PRAT protein subfamilies, namely HP20 and HP30, each comprising two closely related members, could accomplish a role in amino acid and/or chloroplast protein transport.

**Figure 8:**
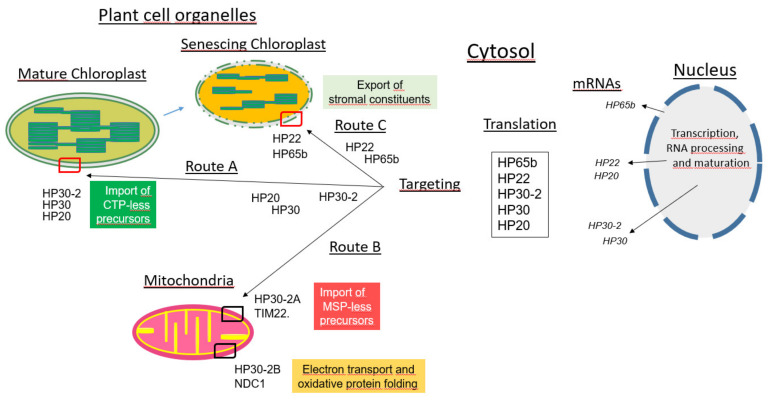
Cartoon summarizing the role of the different PRAT proteins analyzed *in vivo*. HP20, HP22, HP30 and HP30-2 are PRAT proteins that are all encoded in the nucleus. Their genes transcribed depending on developmental and environmental factors. The resulting transcripts undergo processing and modifications and are then transported into the cytosol where they bind to 80 ribosomes and are translated into protein. In turn, HP20, HP22, HP30 and HP30-2 are targeted to different destinations, all four being transported to chloroplasts, whereas a fraction of HP30-2 is dually targeted and thus is also transported to mitochondria. Chloroplast HP20, HP30 and HP30-2 establish a unique protein translocase for the import of chloroplast transit peptide (CTP)-less precursors. Similarly, mitochondrial HP30-2 operates in the import of mitochondrial signal peptide (MSP)-less precursors, while interacting with TIM22 and other components. By contrast, HP22 appears to play a specific role in senescing chloroplasts where it interacts with HP65 and thereby controls the export of stromal proteins to the cytosol. Note that the sizes of the nucleus and different plant cell organelles is not drawn to scale.

Previous expression studies, GFP tagging and biochemical experiments revealed that HP20, HP22, HP30 and HP30-2 are present at different locations in Arabidopsis chloroplasts, HP20 and HP22 being outer plastid envelope proteins and HP30 and HP30-2 being inner plastid envelope proteins [27,28,37,39]. As shown here and elsewhere [37], HP20, HP30 and HP30-2 establish protein complexes involved in the import of transit sequence-less protein such as chloroplast envelope quinone oxidoreductase homologue ceQORH into Arabidopsis chloroplasts (Figure 8, route A). At the chosen nucleotide concentrations, ceQORH traversed the outer envelope membrane and also interacted with components of the inner envelope membrane [37]. After detergent solubilization, 10 different protein spots were identified and classified to fall into three categories: (i) PRAT proteins such as HP20, HP30 and HP30-2, (ii) proteins of the TOC GTPase superfamily of receptors, such as TOC120, TOC90 and TOC34 (42–44), and (ii) molecular chaperones of the heat-shock protein HSP93-V and heat shock cognate protein HSC70 families [45,46], and (Figure 1) [37,38,39]. In addition, with TIC40 and TIC55 (Figure 1) two inner plastid envelope proteins were identified as interacting with ceQORH that have established roles in chloroplast protein import (Table S1). The results presented in Figure 1 are in accordance with crosslinking studies using Elman’s reagent (5,5′-dithiobis(2-nitrobenzoic acid, DTNB) and fractionation experiments [37,39]. Together, compelling evidence was obtained for the presence of a unique translocase in chloroplasts, acting partially in cooperation, partially in parallel to the well-known TOC/TIC system. The specificity of the new system of translocase for transit sequence-less precursors is reminiscent of that of the mitochondrial TIM22 complex in the uptake of proteins lacking cleavable mitochondrial signal peptides [21].

Physiological tests uncovered a slight delay in greening that occurred when etiolated *Athp20;1* and *Athp20;2* seedlings were exposed to white light (Figure S5). This effect was confined to the early hours of seedling de-etiolation and correlated with reduced amounts of nucleus-encoded photosynthetic proteins (Figure S6), at first glance suggesting an impairment of protein import and/or amino acid uptake to happen in *Athp20;1* and *Athp20;2* plants. However, these effects were seen only on growth medium containing sucrose which has documented repressive effects on chloroplast development [54,55]. In fact, no delay in greening occurred in *Athp20;1* and *Athp20;2* seedlings illuminated at high light intensities in the absence of sucrose. Seedling viability tests on sucrose-free medium did not reveal increased light sensitivities of the *Athp20;1* and *Athp20;2* mutants as compared to the wild-type (Figure S8). Similarly, *Athp22;2* single and *Athp20::Athp22* double mutants as well as HP22 overexpressing plants had no greening defects (Figure 4 and Figure S10), excluding devastating effects of the mutations on chloroplast integrity and function.

Arabidopsis mutants lacking HP30 were viable as well and unaffected in chloroplast biogenesis and greening (Figures S5 and S8). Western blotting carried out with antibodies against plastid- and nucleus-encoded plastid proteins did not reveal major differences for greening *Athp30;2* and *Athp30;3* seedlings (Figure S7). Amino acid uptake experiments and pulse-labelling studies with ^35^S-methionine on developing and mature as well as senescing chloroplasts did not indicate significant differences for wild-type, *Athp20;2* and *Athp30;3* plants (Figures S3, S4, S9 and S12). Incorporation rates of ^35^S-methionine declined similarly for all three genotypes over plant development and was highest in young seedlings (Figure S12). In fact, greening seedlings took up more ^35^S-methionine into their plastids than older seedlings and plants and a decline of amino acid incorporation became apparent when mature plants entered the senescence program (Figure S12). These effects were indiscernible for wild-type, *Athp20;2* and *Athp30;3* plants (Figure S12) and thus excluded a role of HP20 and HP30 as plastid bulk amino acid transporters.

### 3.2. Role of Mitochondrial HP30-2 in planta

HP30-2 is present both in chloroplasts and mitochondria [19,28,37]. As shown here and elsewhere [28], HP30-2 interacts with at least 10 different proteins in mitochondria (Figure 2 and Figure 3). HP30-2 in fact forms two distinct protein complexes in the inner mitochondrial membrane that participate in the import of cytosolic precursor proteins into mitochondria [28]. One main complex, dubbed HP30-2A, contains TIM22, mitochondrial (mt) HSP70 and MIA40 and is involved in the import of mitochondrial signal peptide (MSP)-less precursors into mitochondria (Figure 8, route B). It couples HSP70-dependent protein translocation to MIA40-mediated oxidative protein folding. The other HP30-2 protein complex, dubbed HP30-2B, contains the C subunit of the alternative NAD(P)H dehydrogenase (also called type II NDH) [56,57] as well as mtHSP70. The role of complex HP30-2B is not yet understood but it is attractive to hypothesize that it may control cell viability. Because NDC1 was previously implicated in a non-canonical and non-phosphorylating electron transport chain [58] towards the cytochrome *c* oxidase, it is likely that abolition of HP30-2 and NDC1 function, as encountered in the *Athp30-2* mutants isolated, gave rise to the generation of apoptotic signals causing growth defects, cell death and seedling lethality during greening (this study) and in plants grown under alternate dark-light cycles [27,28].

### 3.3. Role of Chloroplast HP22 during Leaf Senescence

Chloroplast HP22 operates specifically during leaf senesce (Figure 8, route C). Evidence was obtained for a function of HP22 as protein export component (Figure 4 and Figure 5) that may be needed to allow the release of organic matter from the stroma to the cytosol in senescing leaf mesophyll cells. Using transplastomic plants expressing promoter-UTR-driven GFP in chloroplasts, we report on a system that allows following plastid leakage in vivo and *in planta*. While *Athp22;2* plants showed a delay in the leakage of stromal GFP, HP22 overexpressors displayed a markedly increases GFP loss from chloroplasts (Figure 5). Plants overexpressing HP22 in fact entered senescence more rapidly and released significantly more stromal GFP into the cytosol per time unit than wild-type plants (Figure 5). Because no such effects were observed for *Athp20* or *Athp30* plants under conditions of natural and artificial, abscisic acid-induced or methyl jasmonate-induced leaf senescence (Figures S13–S17), these findings define HP22 as new, unique factor controlling the senescence process.

Biochemical experiments additionally showed that HP22 and HP20 establish different protein complexes in the plastid envelope (Figure 6A). HP22 interacts with at least 10 other proteins (Figure S18) of which the protein identified as HP65b [52] showed amino acid sequence homology to the bacterial trigger factor chaperone [53]. Structural and kinetic data published by other groups suggest that bacterial trigger factor traps both partially folded and unfolded polypeptide chains by an encapsulation mechanism that allows a vast range of folded protein structures to be protected from aggregation and mis-folding [59]. Trigger factor binds its target proteins at multiple sites and primarily through hydrophobic interactions. While the initial binding of trigger factor is highly dynamic and short-lived, subsequent steps provide more stable and long-lasting intermediates in which the substrate protein is kept in an extended, unfolded state [60]. It is attractive to assume that such unfolded polypeptide conformation may be needed to allow stromal proteins to pass the plastid envelope which is normally impermeable for folded polypeptide chains. By virtue of the joint action of HP22 and HP65b, in fact, a relay could be established to permit the export of chloroplast proteins from the stroma to the cytosol in senescing plants. It is not clear yet in which conformation GFP and the other tested stromal proteins (LSU, EF-T) left senescing chloroplasts. Given the pore-forming properties of HP22 it seems likely that proteins to be transported via HP22 would need to unfold before being exported. For GFP, some refolding then would need to occur in the cytosol, allowing its fluorescence detection by confocal laser scanning microscopy. In fact, GFP has routinely been used for studying protein import into chloroplasts such that the postulated unfolding/refolding mechanisms appears to be functional. Whether mitochondrial HP22 (Figure 6B) can establish a similar system of protein export from mitochondria, remains to be established in future work.

Genetic studies showed that depletion of HP22 or HP65b function provoked significant delays in leaf senescence progression and chloroplast decay (Figure 7). In fact, *Athp65b-RNAi-1#2* and *Athp22;2* plants retained longer total and soluble chloroplast proteins than wild-type plants. When *Athp65b-RNAi-1#2 RNAi* plants were crossed with the *Athp22;2* mutant, a double mutant (*Athp22;2::Athp65b-RNAi-1#2)* was generated that lacked both HP65b and HP22 and had an even stronger senescence phenotype than each parental genotype (Figure 7). *Athp22;2::Athp65b-RNAi-1#2* plants contained high levels of total and soluble chloroplast proteins and maintained these levels significantly longer than wild-type plants (Figure 7). Together these findings suggest HP22 and HP65b to be part of a common mechanism controlling chloroplast protein export during leaf senescence in Arabidopsis.

## 4. Materials and Methods

### 4.1. Arabidopsis Mutants and RNAi Lines

Knock-out mutants referred to as *Athp20;1* and *Athp20;2* (SALK_020671 and SALK_125640), *Athp30;2* and *Athp30;3* (SALK_112126 and SALK_046194), *Athp30-2;1* and *Athp30-2;2* (SALK_136524 and SALK_ 136525), as well as *Athp22;1* and *Athp22;2* (SALK_001823 and SALK_047513) were obtained from the Salk Institute Genomic Analysis Laboratory collection [47] and Gabi Kat collection [50]. Identification of homozygous mutant plants was achieved by polymerase chain-reaction-based techniques [48], using appropriate primers [37]. Isolation and characterization of the *Athp65b* mutants and RNAi lines is described in the SI section.

### 4.2. Plant Growth Conditions

All plants were grown at 23 °C under long-day conditions (16 h at 100 μE m^−2^ s^−1^ light, 8 h dark). For illumination experiments, seeds were surface-sterilized with 70% (v/v) ethanol/0.1% (v/v) Tween 20 and germinated on half-strength Murashige-Skoog-agar medium containing or lacking 1% (w/v) sucrose for 4–5 days in the dark and exposed to continuous white light of 30–40 µE m^−2^ s^−1^ (referred to as low-light conditions) or ≈125 µE m^−2^ s^−1^ (referred to as high-light conditions) [61,62]. For photo-bleaching tests, seedlings were grown on media lacking sucrose and exposed to white light of ≈210 µE m^−2^ sec^−1^ [61,62]. Plant hormone treatments on mature plants were carried out as described [63].

### 4.3. Seedling Viability Tests

Seedling viability was assessed by tetrazolium staining [64]. For statistic assessment, pools of about 250 seeds were analyzed in three replicate experiments.

### 4.4. Pulse-Labeling of Total Leaf Proteins with 35S-Methionine

If not stated otherwise, pulse-labeling of total leaf proteins was performed with ^35^S-methionine (37 TBq/mmol, Amersham-Pharmacia) for 2 h prior to harvest [63,65]. After incubation, protein was extracted with either a mixture of 80% (v/v) acetone/20% (v(v) SDS sample buffer (2.9% SDS, 68 mM Tris-HCl, pH 6.8, 10% (v/v) glycerol, 0.1 M β-mercaptoethanol) [66] or buffer A (50 mM Tris-HCl, pH7.8, 25 mM KCl, 10 mM MgCl, 1 mM PMSF, 1 mM NaF, 0.5% (v/v) mercaptoethanol, 1% (v/v) Triton X-100, 250 mM sucrose) [67], followed by further homogenization in a Branson Sonifier (model B-12, microtip, 80 W, 1 min) and precipitation with 5% (v/v) trichloroacetic acid. Protein that had been extracted with acetone/SDS sample buffer was cleared by centrifugation and only the supernatant was used for SDS-PAGE. Protein that had been extracted with buffer A and precipitated with trichloroacetic acid was washed with acetone, ethanol and ether, dried and dissolved in SDS sample buffer. Protein was separated electrophoretically as described before. Immunodetection of proteins was carried out using the indicated antisera (Agrisera, Vännäs, Sweden) and either an enhanced chemiluminescence system (ECL, Amersham-Pharmacia) or anti-rabbit, anti-goat, alkaline phosphatase system with 4-nitroblue-tetrazoliumchloride (NBT) and 5-bromo-4-chloro-3-indolyl phosphate (BCIP) [68].

### 4.5. Chloroplast Isolation, Amino Acid Uptake and In Organello-Protein Synthesis

Chloroplasts were isolated from seedling or leaf homogenates by density gradient centrifugation on Percoll (Pharmacia LKB Biotechnol. AB, Sweden) [69]. Re-isolated, intact plastids were suspended in import buffer lacking ATP and energy depleted [70]. Uptake of ^35^S-methionine and ^14^C amino acids into isolated chloroplasts was monitored by using the filter paper disk method of Mans and Novelli [71]. For in organelle protein synthesis, chloroplasts were incubated for 2 h with [^35^S]-methionine (1.87 MBq per 50-µl assay, 37 TBq/mmol; Amersham Pharmacia, Uppsala, Sweden) in a buffer containing 50 mM HEPES-KOH, pH 8.0, 40 µM of each proteinogenic amino acids except for L-methionine, 10 mM dithiothreitol, 5 mM Mg-ATP, 10 mM MgCl_2_, and 0.35 M sucrose [63,65]. Labeling was stopped by collecting intact plastids by centrifugation or directly adding an equal volume of doubly-concentrated SDS sample buffer [63,65].

### 4.6. Isolation of Plastid Envelope Protein Complexes

The chloroplast envelope quinone oxidoreductase homologue (ceQORH) was used as a model for a transit sequence-less cytosolic precursor [40]. It was expressed as ^35^S-methionine-labeled, hexa-histidine ([His)_6_)-tagged fusion with or without GFP (ceQORH-GFP-(His)_6_) in *E. coli* strain SG13009 (Qiagen) as described [35,40]. By analogy, (His)_6_-tagged versions of HP20 and HP22 were prepared and purified. In each case, the protein extract then was diluted to yield urea concentrations of 0.2 M and subjected affinity chromatography on Ni-NTA agarose. The final, ≈90% pure proteins were incubated with isolated, energy-depleted Arabidopsis plastids in the presence of 0.1 mM Mg-GTP and either 0.1 mM Mg-ATP (ceQORH) or 2.5 mM Mg-ATP (HP20 and HP22) for 15 min. After this time, intact plastids were recovered on Percoll, lysed under hypertonic conditions, and total membranes isolated as described [41]. After solubilization of these membranes with 2% Triton X100 in buffer containing 50 mM Tris-HCl, pH 7.5, 300 mM NaCl, 20 mM imidazole-HCl, pH 8.0, and 1 mM PMSF [41], and a step of centrifugation at 100,000 g for 15 min, proteins interacting with ceQORH, HP20 and HP22, respectively, were identified by either 1D-SDS-PAGE on 10–20% polyacrylamide gels or 2D-SDS-PAGE including isoelectric focusing in the first dimension and SDS gradient gel electrophoresis in the second dimension [69,72]. Protein was stained with Coomassie Brilliant Blue G25 and subjected to sequencing [73].

### 4.7. Plastid Leakage Assay

For testing plastid integrity in planta, transplastomic plants expressing a Sl-pRC32 (rrn16::accD*)-GFP constructs [51] in the wild-type, Athp22;2 mutant or HP22-overexpressing backgrounds were used. For the detection of GFP fluorescence, the excitation wavelength was 488 nm and the barrier filter BP 530 (band pass, 515–545 nm) was used. For monitoring chlorophyll fluorescence, the excitation wavelength was 568 nm and the barrier filter BP 590 (long pass, >590 nm) was used [51]. Alternatively, plastids were isolated from leaf homogenates by centrifugation on Percoll/sucrose gradients, and stromal marker proteins, such as the large subunit of ribulose-1,5-bisphosphate carboxylase/oxygenase (LSU) and elongation factor EF-Tu, as well as thylakoid markers, such as the light-harvesting chlorophyll a/b binding proteins of photosystem II, quantified on an equal plastid number basis as described [63], using specific antisera and either enhanced chemiluminescence (ECL)-based (Amersham) or anti-rabbit, anti-goat, alkaline phosphatase-based detection systems. For testing plastid integrity in vitro, sedimentation analyses were carried out according to van Leyen et al. [74]. Briefly, plastids that had been isolated on Percoll were resuspended in buffer and kept for 2 h in darkness before being pelleted by centrifugation. Protein contained in the plastid and supernatant fractions were precipitated with trichloroacetic acid, washed with acetone and ethanol and subjected to SDS-PAGE.

### 4.8. Analysis of Mitochondrial Membrane Proteins

Mitochondria were isolated from leaf homogenates as described [75] and incubated with carboxy-terminally, hexa-histidine (His)_6_-tagged, ^35^S-HP30-2 or ^35^S-HP22 that had been produced in *E. coli* from respective cDNAs and purified to apparent homogeneity by Ni-NTA affinity chromatography (see above). After incubation for 30 min at 23 °C, intact mitochondria were re-isolated and fractionated [75]. Outer or inner mitochondrial membranes were solubilized with 1% (v/v) or 3.5% (v/v) digitonin in a buffer containing 30 mM HEPES-KOH, 200 mM K-acetate, 1 mM EDTA, and 1 mM EGTA (pH 7.4) [76]. Insoluble membrane components were removed by centrifugation at 109,000 × g in a Beckman TL100 ultracentrifuge, TLA45 rotor, for 30 min. Mitochondrial membrane proteins interacting with HP-30-2-(His)_6_ or HP22-(His)_6_, respectively, were identified by SDS-PAGE (see above) and Coomassie or silver staining, Western blotting with specific antibodies or protein sequencing. For total protein analyses, outer and inner mitochondrial membranes were processed as described [76].

### 4.9. Other Techniques

Chlorophyll measurements were performed according to Porra et al. [77]. Scanning Transmission Electron Microscopy (STEM) was carried out in a Philips ESEM XL 30 microscope [33].

## 5. Conclusions

The HP22- and HP65b-mediated protein export mechanism from chloroplasts discovered here appears to be just one way of liberating organic matter to the cytosol. Still, many of its molecular details need to be explored. Another pathway involves 13-lipoxygnase (13-LOX) that catalyzes the *regio*- and *stereo*-specific oxidation of unsaturated membrane fatty acids such as α-linolenic acid and thereby introduces holes into the plastid envelope [33]. Other well-characterized pathways comprise autophagy, senescence-associated vacuoles, and chloroplast vesiculation and were implicated in bulk breakdown of organelles, degradation of stromal proteins such as RuBisCO, and transfer of mostly envelope membrane as well as thylakoid membrane proteins to lytic vacuoles [78,79,80]. It will be interesting to see whether HP22 is part of one of these pathways or may act independently. Work is needed to dissect these pathways genetically given that biochemical approaches may fail because of the observed instability of the membrane protein assemblies *in planta* during leaf senescence.

## Data Availability

Data is contained within the article and respective supplementary materials.

## Supplementary Materials

The following are available online at https://www.mdpi.com/article/10.3390/plants10050958/s1, Figure S1: Characterization of the T-DNA insertion lines Athp20;1 (SALK_020671) and Athp20;2 (SALK_125640, Figure S2: Characterization of the T-DNA insertion lines Athp30;2 (SALK_112126) and Athp30;3 (SALK_046194), Figure S3: Phenotypic and cell biological characterization of light-grown Athp20 and Athp30 knock-out mutant plants, Figure S4: Protein patterns of light-grown Athp20;2 and Athp30;2 plants, Figure S5: Greening of etiolated Athp20 and Athp30 seedlings under low-light conditions, Figure S6: Protein accumulation patterns in Athp20 mutant seedlings during greening under low-light conditions, Figure S7: Protein accumulation patterns in Athp30 mutant seedlings during greening, Figure S8: Seedling viability of Athp20 and Athp30 mutant seedlings during greening, Figure S9: Amino acid uptake into isolated plastids during plant etiolation and greening, Figure S10: Characterization of Ath20::Athp22 double mutants, Figure S11: Characterization of Ath20 and Athp22 as well as Ath20::Athp30 double mutants, Figure S12: 2 Amino acid uptake into isolated plastids during leaf senescence of wild-type (WT) versus Athp20;2 and Athp30;3 mutant plants, Figure S13: Leaf phenotype of wild-type and Athp20 and Athp30 mutant plants during senescence, Figure S14: Chloroplast protein pattern of wild-type (WT) versus Athp20;2 and Athp30;3 mutant plants during natural and artificial leaf senescence, Figure S15: Protein accumulation patterns in A. thaliana wild-type and mutant Athp20;2 during senescence, Figure S16: Protein accumulation patterns in A. thaliana wild-type and mutant Athp30;3 during senescence, Figure S17: Chloroplast protein pattern of wild-type (WT) versus Athp20;2 and Athp30;3 mutant plants during dark-induced senescence, Figure S18: Identification of proteins interacting with HP20 (A) and HP22 (B) in chloroplasts, respectively, Figure S19: Identification and characterization of RNAi plants lacking HP65b, Figure S20: Analysis of HP65b and HP22 expression over the course of plant development, Table S1: Chlorophyll accumulation in Athp20 and Athp30 mutant seedlings during greening, Table S2: Uptake of 35S-methionine into isolated plastids during plant etiolation and greening, Table S3: Amino acid uptake into isolated plastids during leaf senescence of wild-type versus Athp20;2 and Athp30;3 mutant plants.

## Abbreviations

GFP	green fluorescent protein
HP	hypothetical protein
PRAT	preprotein and amino acid transporter
TIC	translocase of the inner envelope of chloroplasts
TOC	translocase of the outer envelope of chloroplasts
