# Supplementary material for: PRAT Proteins Operate in Organellar Protein Import and Export in Arabidopsis thaliana
Source: Plants (Basel). 2021 May 11;10(5):958. doi: 10.3390/plants10050958 (PMC8151980; doi:10.3390/plants10050958)
Supplement: Supplementary file 1 [file plants-10-00958-s001.zip › plants-1191367-supplementary.pdf]

PRAT Proteins Operate in Organellar Protein Import and Export in *Arabidopsis thaliana*

Claudia Rossig [1], John Gray [2], Oscar Valdes [1], Armin Springer [3], Sachin Rustgi [4,5], Diter von Wettstein [5,‡], Christiane Reinbothe [1], Joachim Rassow [6] and Steffen Reinbothe [1,*]

1. Laboratoire de Génétique moléculaire des Plantes, Université Grenoble-Alpes, BP53F, 38041 Grenoble, France; claudia.rossig@gmx.de (C.R.); oscar.valdes@myubt.de (O.V.); bt434375@myubt.de (C.R.)
2. Department of Biological Sciences, University of Toledo, 2801 West Bancroft Street, OH 43606 Toledo, USA; jgray5@utnet.utoledo.edu
3. Medical Biology and Electron Microscopy Centre, University Medical Center Rostock, Strempelstraße 14, 18057 Rostock, Germany; armin.springer@med.uni-rostock.de
4. Department of Plant and Environmental Sciences, Pee Dee Research and Education Center, Clemson University, SC 29506 Florence, USA; srustgi@clemson.edu
5. Department of Crop and Soil Sciences, Washington State University, 99164 Pullman WA, USA
6. Department of Cell Biochemistry, Institute for Biochemistry and Pathobiochemistry, Ruhr-University Bochum, Universitätsstrasse 150, 44780 Bochum, Germany; joachim.rassow@ruhr-uni-bochum.de

‡ Deceased 13 April 2017
* Correspondence: sreinbot@ujf-grenoble.fr; Phone: 0033 476 63 5418; Fax: 0033 476 63 5418

1. SI Materials and Methods

1.1. Isolation and characterization of Athp65b mutants and RNAi lines

Athp65b mutant seeds (SALK_089907, SALK_095609, SALK_062664) were obtained from the Salk Institute Genomic Analysis Laboratory collection [1] and homozygous mutants identified by a polymerase chain-reaction (PCR)-based approach [2], using appropriate primer combinations [3,4]. For the construction of inverted repeats that give rise to double-stranded RNA and induce the directed degradation of mRNA, published protocols were used [5,6]. Constructs with a hairpin-loop forming intron (PDK, pyruvate orthophosphate dikinase) between sense and antisense gene fragments were created with the help of the vector pHannibal [5]. The gene fragments were synthesized by PCR [2] using appropriate primers, with added restriction sites defining the final orientation (sense/antisense) after cloning. The generated construct was transferred into the binary vector pArt27 [6].

1. Alonso JM, Stepanova AN, Leisse TJ, Kim CJ, Chen H, Shinn P, Stevenson DK, Zimmerman J, Barajas P, Cheuk R *et al.* (2003) Genome-wide insertional mutagenesis of *Arabidopsis thaliana*. *Science* **301**, 653-657.
2. Innis MA, Gelfand DH, Sninsky JJ, White TJ (1990) *PCR Protocols* (Academic Press, San Diego, CA).
3. Ruiz-Ferrer V, Voinnet O (2009) Roles of plant small RNAs in biotic stress responses. *Annu Rev Plant Biol* **60**, 485-510.
4. Kovács-Bogdán E, Soll J, Bölter B (2010) Protein import into chloroplasts: the Tic complex and its regulation. *Biochim Biophys Acta* **1803**, 740-747.
5. Wessley VS, Helliwell C, Smith NA, Wang MB, Rouse D *et al.* (2001) Construct design for efficient, effective and high-throughput gene silencing in plants. *Plant J* **27**, 581-590.

2. Supplementary Results

2.1. Supplementary Figures

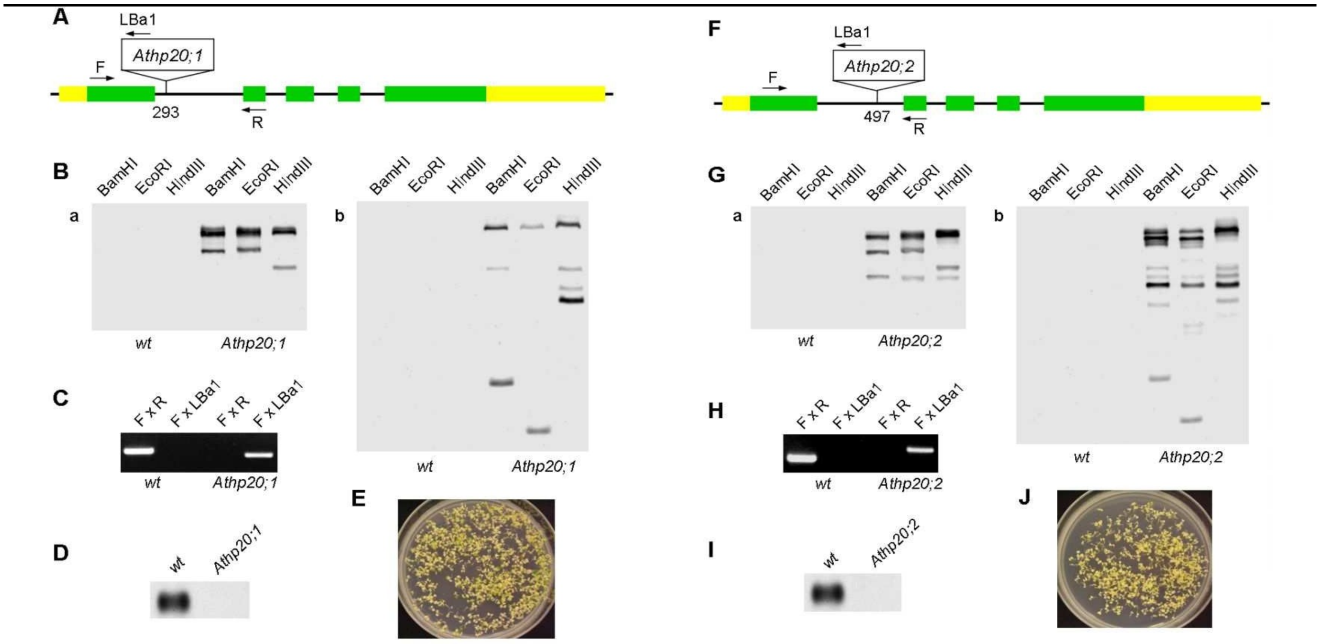

Figure S1. Characterization of the T-DNA insertion lines *Athp20;1* (SALK_020671) and *Athp20;2* (SALK_125640; back-crossed once with Table 20. gene indicating the exact position of the T-DNA insertions as determined by sequencing PCR products amplified with primers F and LBa1. (**B**) and (**G**), Southern blot analysis showing the number of T-DNA insertions by probing against the kanamycin resistance gene of Salk T-DNA (**a**) and a fragment of the left border (**b**) after digestion of 10 µg genomic DNA with *Bam*HI, *Eco*RI and *Hind*III. C and H, PCR- genotyping demonstrating the homozygosity of the mutants by absence of the wild-type allele (primers F x R). Primers F and R correspond to HP20PF and HP20PR. The sizes of the products are 610 bp (R x F, only obtained with wild-type DNA) and 547 bp or 727 bp (F x LBa1, only obtained with mutant DNA). (**D**) and (**I**), Northern blot analysis to detect *HP20* transcripts using mRNA isolated from 3 weeks-old plants. (**E**) and (**J**), Growth behaviour on selective MS agar containing kanamycin.

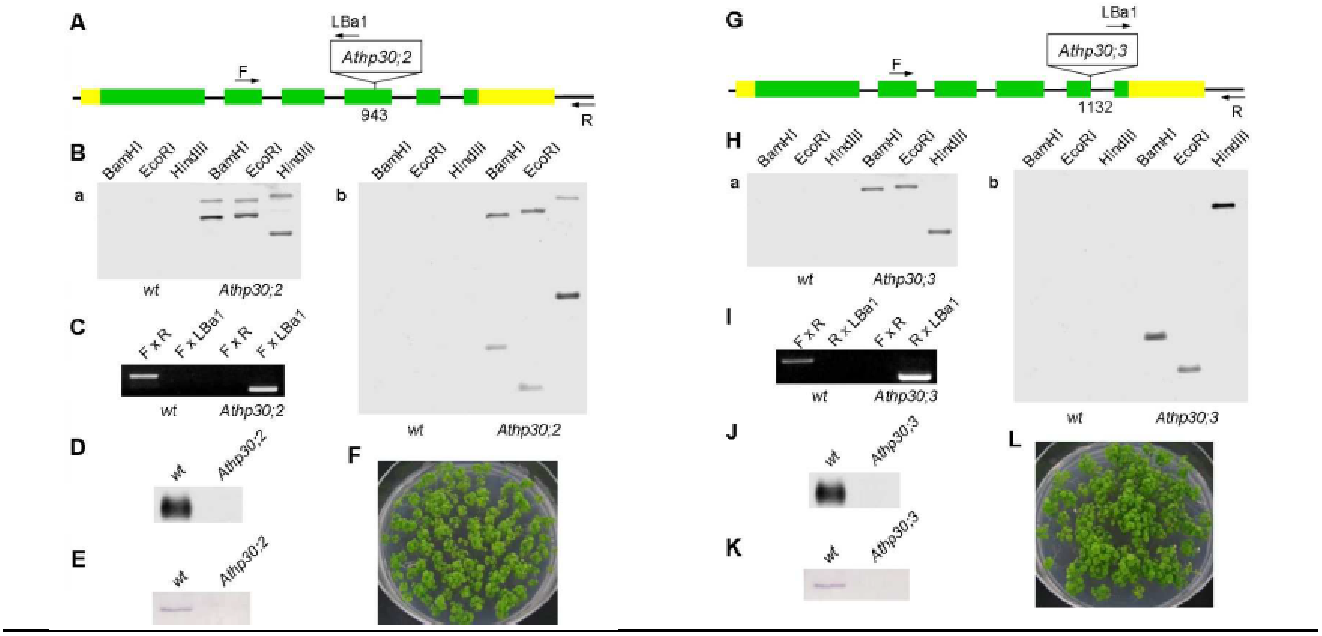

Figure S2. Characterization of the T-DNA insertion lines *Athp30;2* (SALK_112126) and *Athp30;3* (SALK_046194). (**A**) and (**G**), Schematic presentation of the *HP30* gene indicating the exact position of the T-DNAs as determined by sequencing PCR products amplified with primers F/R and LBa1. (**B**) and (**H**), Southern blot analysis showing the number of T-DNA insertions by probing against the kanamycin resistance gene of Salk T-DNA (**a**) and a fragment of the left border (**b**) after digestion of 10 µg genomic DNA with *Bam*HI, *Eco*RI and *Hind*III. C and I, PCR-genotyping demonstrating the homozygosity of the mutants by absence of the wild-type allele (primers F x R). Primers F and R correspond to HP30GT1 and HP30GT2. The sizes of the products are 1016 bp (RxF, only obtained with wild-type DNA) and 684 bp or 747 bp (FxLBa1, only obtained with mutant DNA). (**D**) and (**J**), Northern blot analysis to detect *HP30* transcripts using mRNA isolated from 3 weeks-old plants. E and K, Western blot analysis of total leaf extracts (40 µg protein/lane) of 3 weeks-old plants and anti-HP30 antibodies. F and L, Growth behaviour on selective MS agar containing kanamycin.

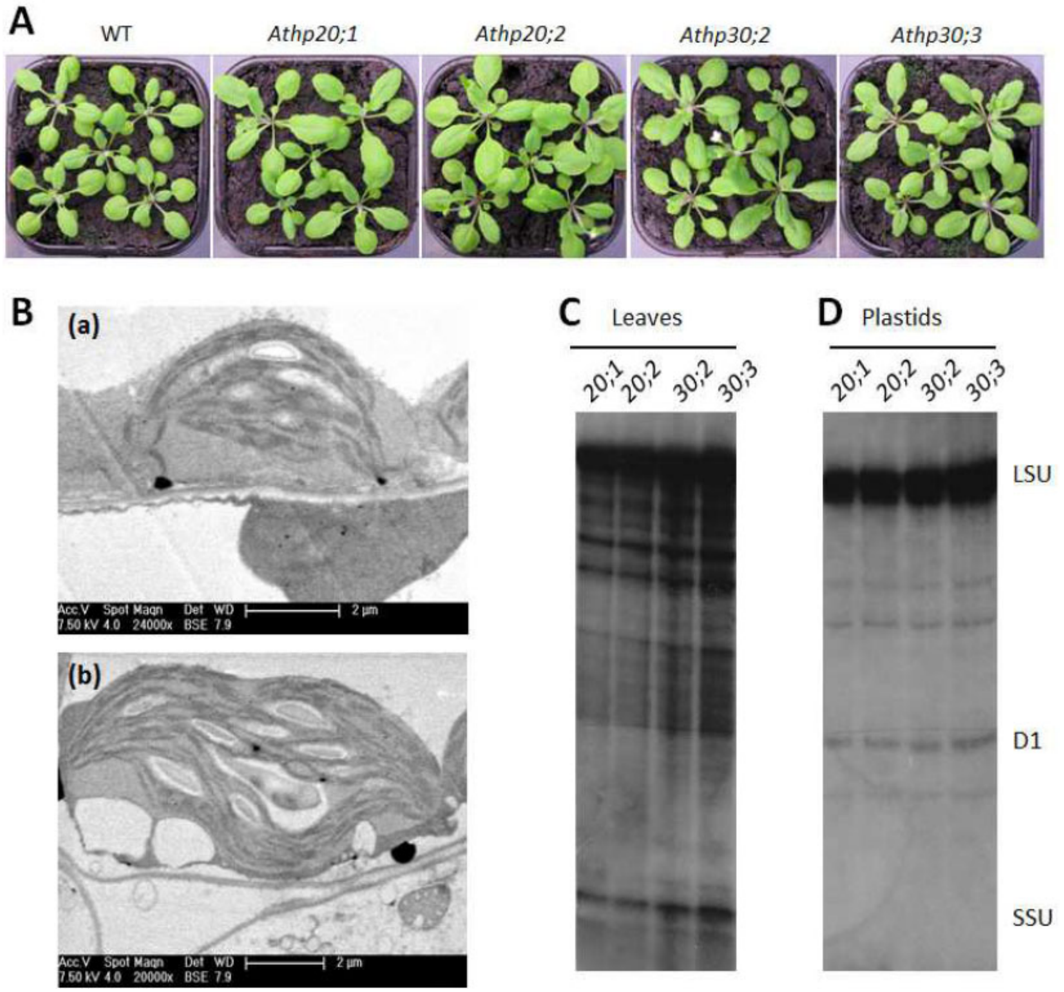

Figure S3. Phenotypic and cell biological characterization of light-grown *Athp20* and *Athp30* knock-out mutant plants. (**A**) Phenotype of 5 weeks-old wild-type versus *Athp20* (*20;1* and *20;2*) and *Athp30* (*30;2* and *30;3*) mutant plants. (**B**) Chloroplast ultrastructure in *Athp20;1* (**a**) and *Athp30;2* (**b**) mutant plants. (**C**) Pattern of total leaf proteins in *Athp20* (*20;1* and *20;2*) and *Athp30* (*30;2* and *30;3*) mutant plants after pulse-labeling with ^{35}S-methionine. (**D**) Patterns of proteins pulse-labeled with ^{35}S-methionine in isolated chloroplasts of *Athp20* (*20;1* and *20;2*) and *Athp30* (*30;2* and *30;3*) mutant plants. LSU and SSU define the large and small subunits of ribulose-1,5-bisphosphate carboxylase/oxygenase; D1 stands for the D1 protein of photosystem II.

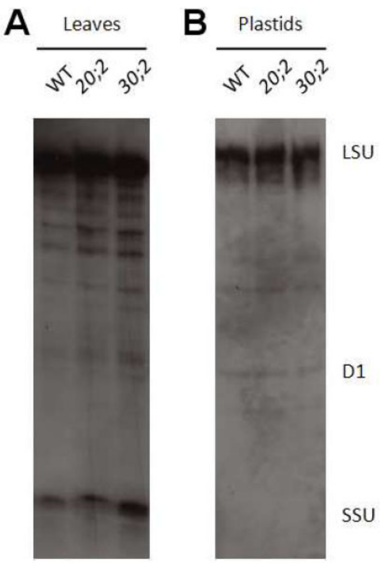

Figure S4. Protein patterns of light-grown *Athp20;2* and *Athp30;2* plants. (**A**) Pattern of total leaf proteins in wild-type (WT) versus *Athp20;2* and *Athp30;2* plants after pulse- labelling with ^{35}S-methionine. (**B**) Patterns of proteins pulse-labelled with ^{35}S-methionine in isolated chloroplasts of (WT) versus *Athp 20;2* and *Athp30;2* plants.

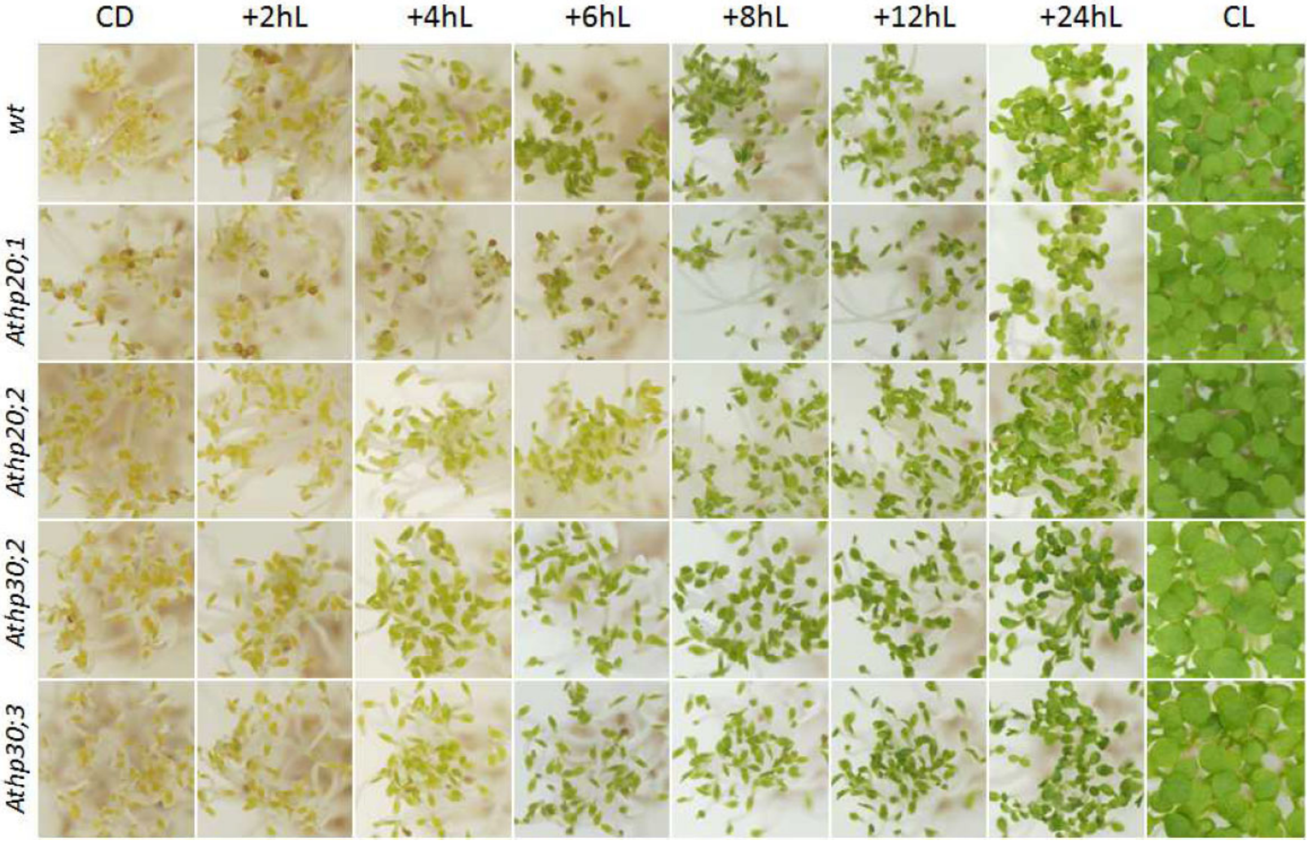

Figure S5. Greening of etiolated *Athp20* and *Athp30* seedlings under low-light conditions. Wild-type and *Athp20* and *Athp30* knock-out mutant seedlings were grown in the dark for 4.5 days and exposed to white light at 40–50 µE m^{-2} s^{-1} (low-light) for the indicated time periods (in h). Photographs were taken for a representative number of seedlings. CD and CL stand for growth in continuous darkness or continuous light. See also Table S2.

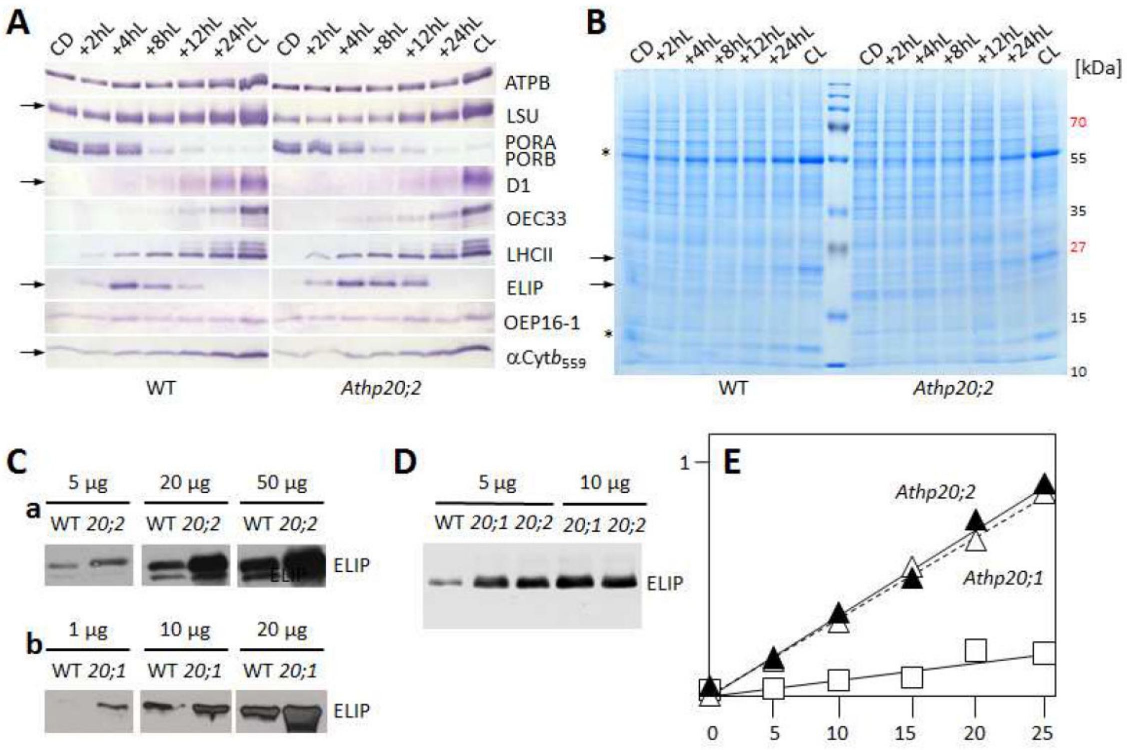

Figure S6. Protein accumulation patterns in *Athp20* mutant seedlings during greening under low-light conditions. (**A**) Western blot analysis of the indicated proteins during greening (in h) of wild-type (WT) and *Athp20;2* mutant seedlings. (**B**) Pattern of Coomassie-stained proteins in wild-type and *Athp20;2* mutant seedlings. **C** as **A** but showing blots containing a serial dilution of protein extracts from etiolated wild-type (WT) versus *Athp20;2* (panel a) and *Athp20;1* (panel b) mutant seedlings after a 4-h white light exposure probed with ELIP antibody. **D**, as **C**, but showing a serial dilution of protein extracts from irradiated wild-type (WT) versus *Athp20;1* and *Athp20;2* mutant seedlings. (**E**) Quantification of protein gel blot data obtained for *Athp20;1* (open triangles) and *Athp20;2* (filled triangles) versus wild-type (WT) (boxes) seedlings. Arrows mark proteins with different expression patterns in wild-type versus mutant seedlings. Asterisks define the large subunits (LSU) and small subunits (SSU) of ribulose-1,5-bisphosphate carboxylase/oxygenase. CD marks protein extracts from dark-grown plants, whereas CL marks protein extracts from light-grown seedlings.

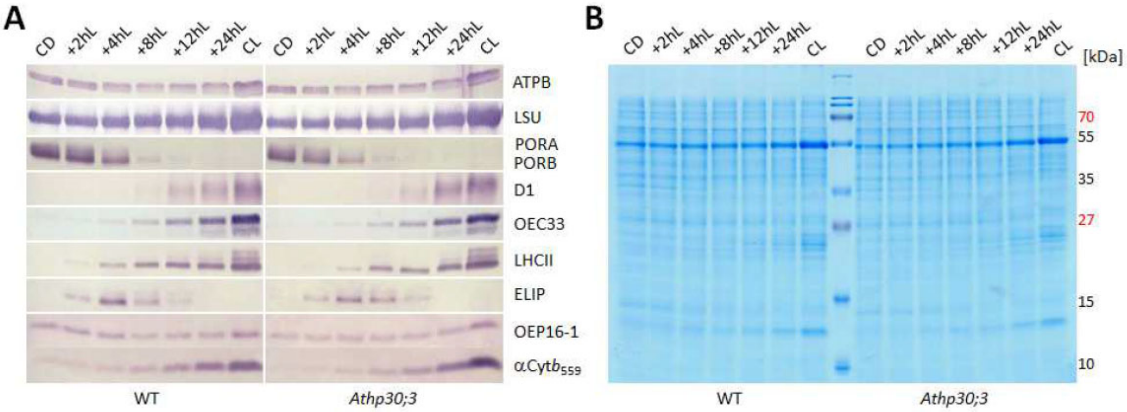

Figure S7. Protein accumulation patterns in *Athp30* mutant seedlings during greening. (**A**) Western blot analysis of the indicated proteins during greening (in h) of wild-type and *Athp30* mutant seedlings. (**B**) Pattern of Coomassie-stained proteins in wild-type and *Athp30* mutant seedlings. CD marks protein extracts from dark-grown plants, whereas CL marks protein extracts from light- grown seedlings.

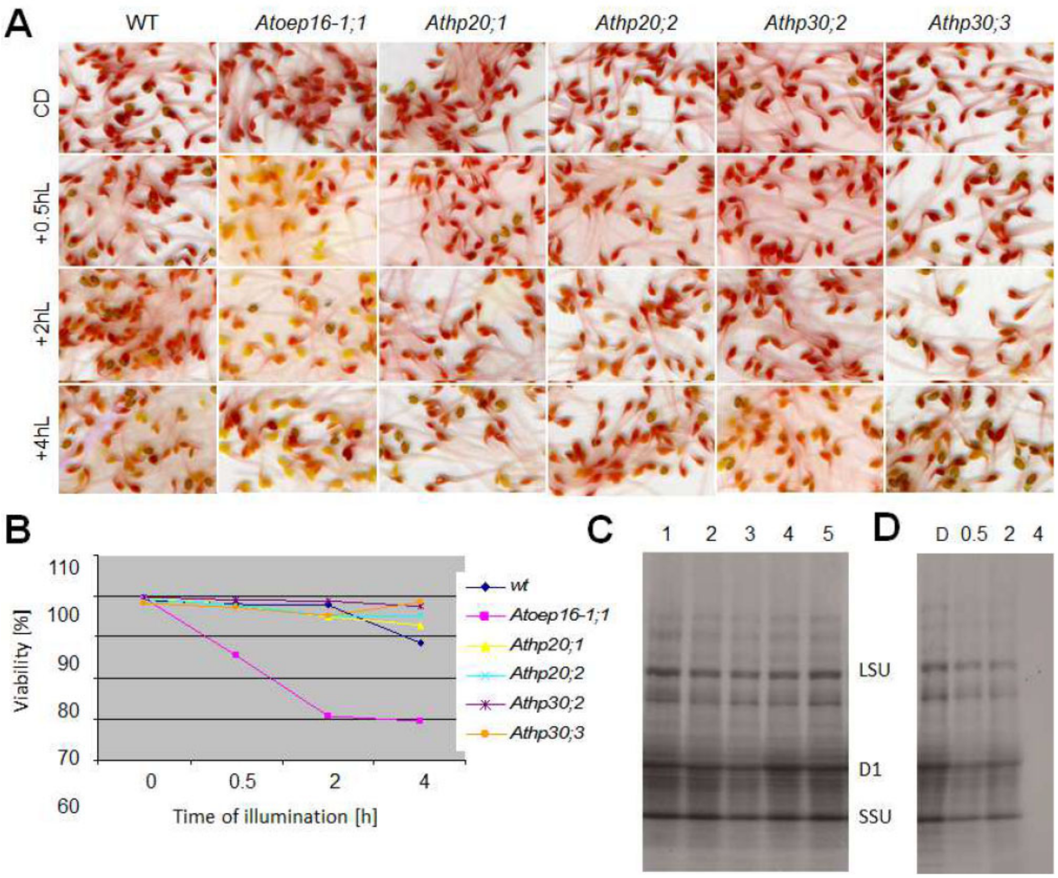

Figure S8. Seedling viability of *Athp20* and *Athp30* mutant seedlings during greening. (**A**) Tetrazolium staining of wild-type versus *Athp20* and *Athp30* mutant seedlings after growth in the dark for 4.5days (CD) and during greening (in h) at 125 µE $m^{-2}s^{-1}$. For comparison, the *Atoep16-1;1* mutant was used. (**B**) Viability of *Atoep16-1;1* versus wild-type, *Athp20* and *Athp30* and mutant seedlings during greening. (**C**) Patterns of total leaf proteins labeled with ^{35}S-methionine in etiolated wild-type (lane 1), *Athp20;1* (lane 2) *Athp20;2* (lane 3), *Athp30;2* (lane 4) and *Athp30;2* (lane 5) seedlings 4 h after the onset of greening. (**D**) Patterns of total leaf proteins labeled with ^{35}S-methionine in etiolated *Atoep16-1;1* seedlings (**D**) and after different time intervals in white light (in h). LSU and SSU indicate the large and small subunits of ribulose-1,5-bisphosphate carboxylase/oxygenase; D1 defines the D1 protein of photosystem II.

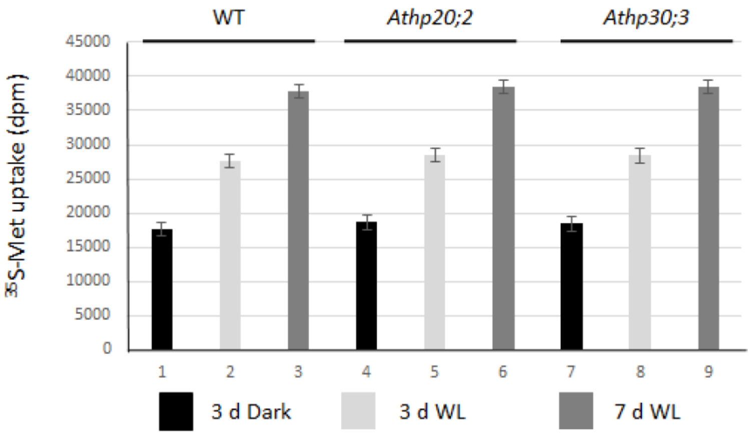

Figure S9. Amino acid uptake into isolated plastids during plant etiolation and greening. Plastid amino acid uptake assays were conducted in the presence of ^{35}S-methionine for wild-type (WT), *Athp20* and *Athp30* seedlings that had been grown for 3 days in darkness (Dark) or for 3 d or 7 d in white light (WL),

respectively. Incorporation rates are given as decays per minute (dpm) and refer to means of three independent experiments. Error bars are indicated. See also Table S3.

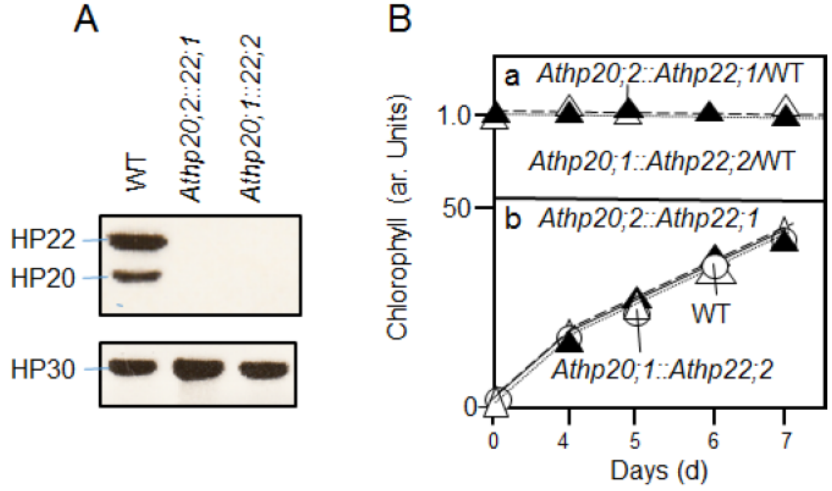

Figure S10. Characterization of *Ath20*::*Athp22* double mutants. (**A**) Western blot analysis to reveal the absence of HP20 and HP22 in the indicated *Athp20::Athp22* double mutants. *Athp20;1* and *Athp20;2* define SALK_020671 and SALK_125640, respectively, whereas *Athp22;1* and *Athp22*;2 define SALK_001823 and SALK_047513, respectively, that were reciprocally used to prepare the indicated double mutants. (**B**) Chlorophyll accumulation in *Athp20;1*::*Athp22*;2 and *Athp20;2::Athp22;1* double mutant (open and filled triangles) after growth in darkness for 4.5 days and subsequent white-light exposures (in hours), as compared to the wild-type (WT) (circles). The lower panel (**b**) shows the kinetics of chlorophyll accumulation, whereas the upper panel (**a**) shows the same data relative to those seen for the wild-type. Note that there are no differences in the kinetics of chlorophyll accumulation detectable for all of the different genotypes and thus the calculated values do not significantly deviate from 1.

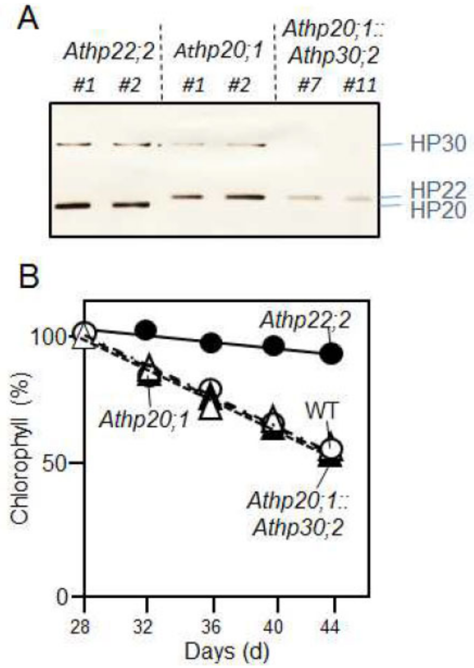

Figure S11. Characterization of *Ath20* and *Athp22* as well as *Ath20*::*Athp30* double mutants. A, Western blot analysis to reveal the absence of HP20 and HP22 in the generated single double mutants, using mono-specific antibodies. *Athp22#1* and *Athp22#*2 define SALK_001823 and SALK_047513, respectively: *Athp30;2* and *Athp30;3* define SALK_112126 and SALK_046194, respectively: and *Athp20;1::Athp30;2* define

double mutants of SALK_020671 and SALK_112126. B, Decay of chlorophyll in 4 weeks-old single *Athp22#2* (dots) and *Athp20;1* (open triangles) mutants as well as A*hp20;1::Athp30;2* double mutants (filled triangles) and wild-type (circles) plants undergoing leaf senescence.

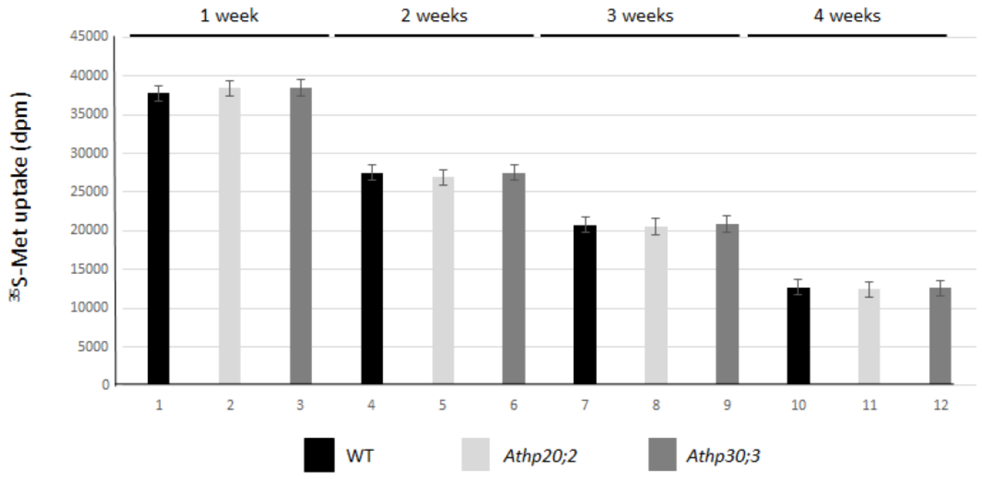

Figure S12. Amino acid uptake into isolated plastids during leaf senescence of wild-type(WT) versus *Athp20;2* and *Athp30;3* mutant plants. Wild-type(WT), *Athp20* and *Athp30* plants were cultivated in alternate 16h light/8h dark cycles for the indicated time periods (weeks) and their chloroplasts isolated on Percoll. Plastid amino acid uptake was measured with ^{35}S-methionine. Rates of amino acid uptake are given as decay per minutes (dpm) and refer to means of three independent experiments. Error bars are indicated. See also Table S4.

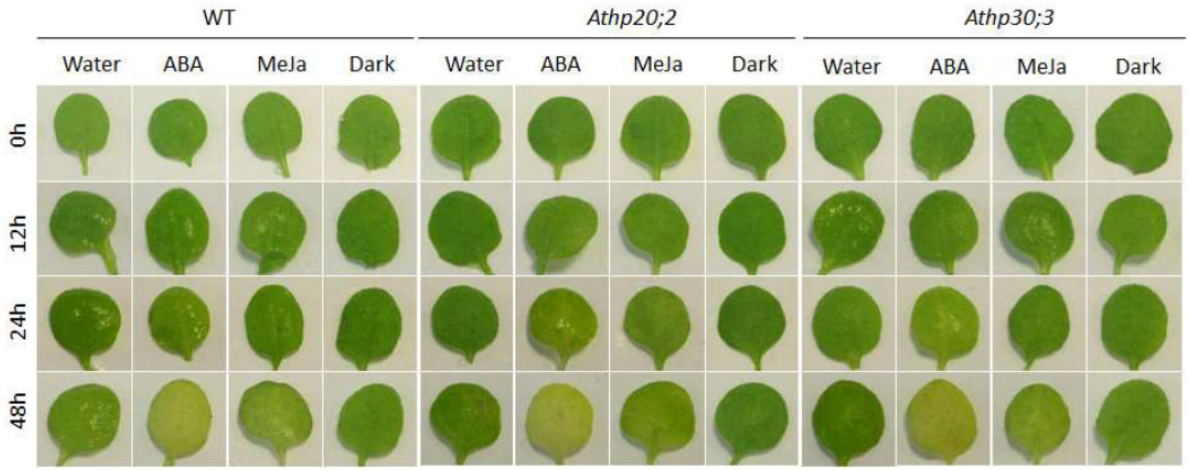

Figure S13. Leaf phenotype of wild-type and Athp20 and Athp30 mutant plants during senescence. Visual inspection of leaves of 3 weeks-old plants of A. thaliana wild-type and mutants Athp20;2 and Athp30;3 that were immersed in tap water, a 0.1 mM ABA solution, a 45 µM MeJA solution and subsequent illumination (40 µE m^{-2} s^{-1}) or subjected to dark treatment for the indicated time. A representative leaf is shown for each treatment.

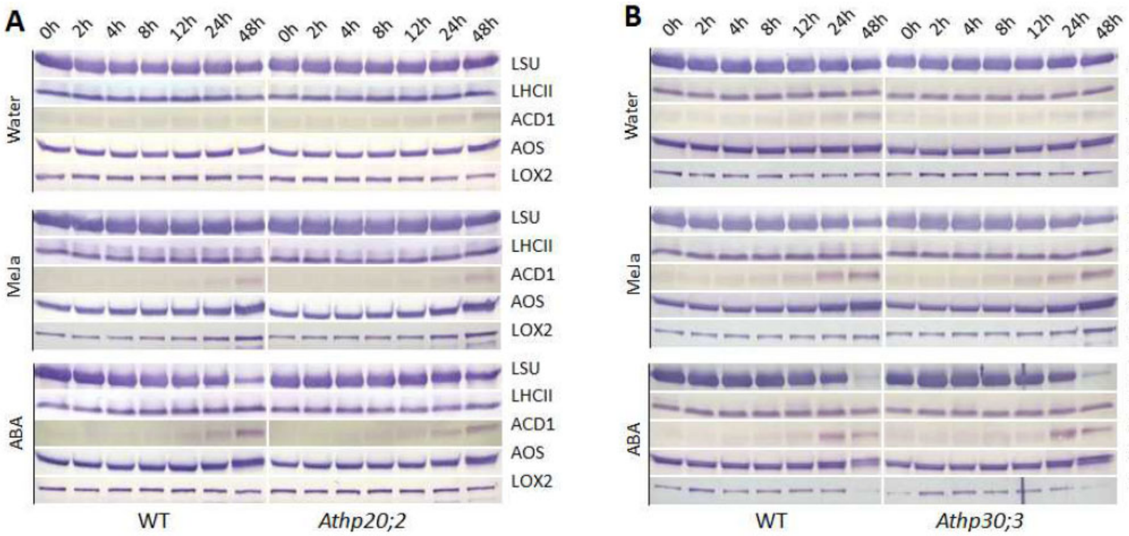

Figure S14. Chloroplast protein pattern of wild-type (WT) versus Athp20;2 and Athp30;3 mutant plants during natural and artificial leaf senescence. (**A**) Protein gel blot analysis of prominent chloroplast markers in 3 weeks-old plants during ABA, MeJA and water treatments. Leaves were cut and incubated in 0.1 mM ABA, 45 µM MeJA or tap water for the indicated time periods. Depending on the specificity of the antisera, amounts of 20 µg (LSU, LHCII and LOX2) and 40 µg (ACD1, AOS) of total leaf proteins, respectively, were subjected to analysis. **B**, as **A**, but depicting the results for Athp30;3 mutant plants.

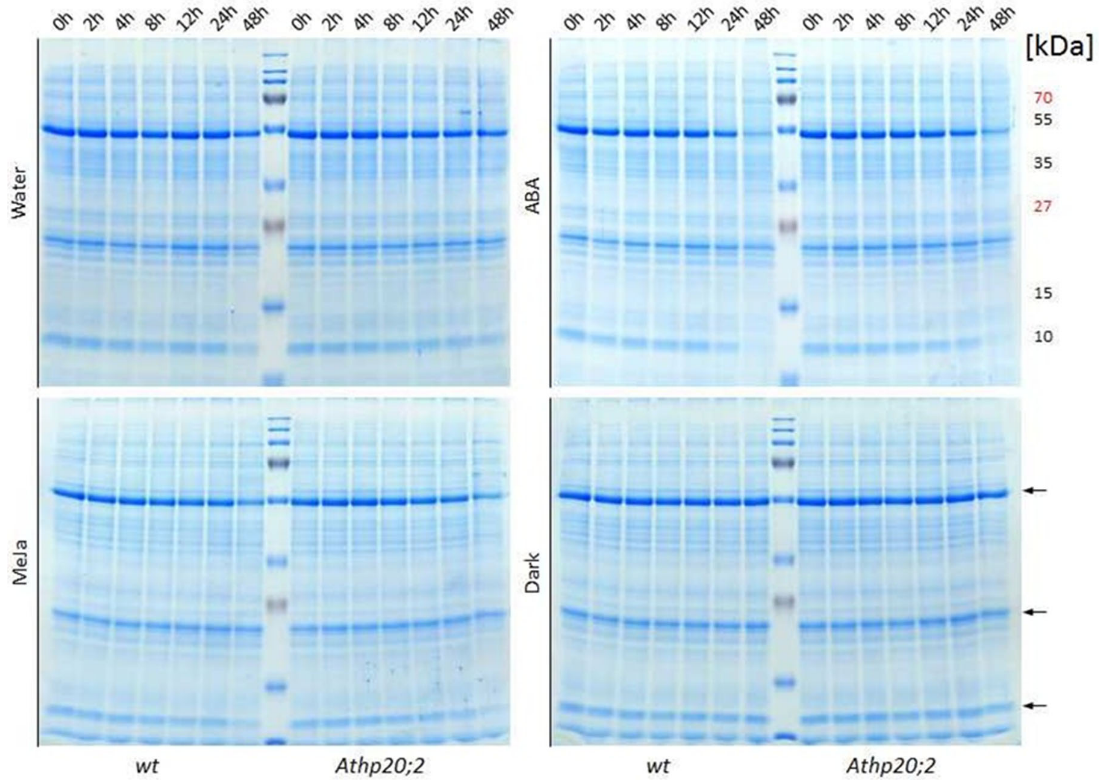

Figure S15. Protein accumulation patterns in A. thaliana wild-type and mutant Athp20;2 during senescence. Leaves were cut and incubated in 0.1 mM ABA, 45 µM MeJA or tap water or kept in continuous darkness for the indicated time periods. Coomassie staining of a representative separation of total proteins (20 µg/lane). The arrows mark LSU (~55 kDa), LHCII (~27 kDa) and SSU (~12 kDa).

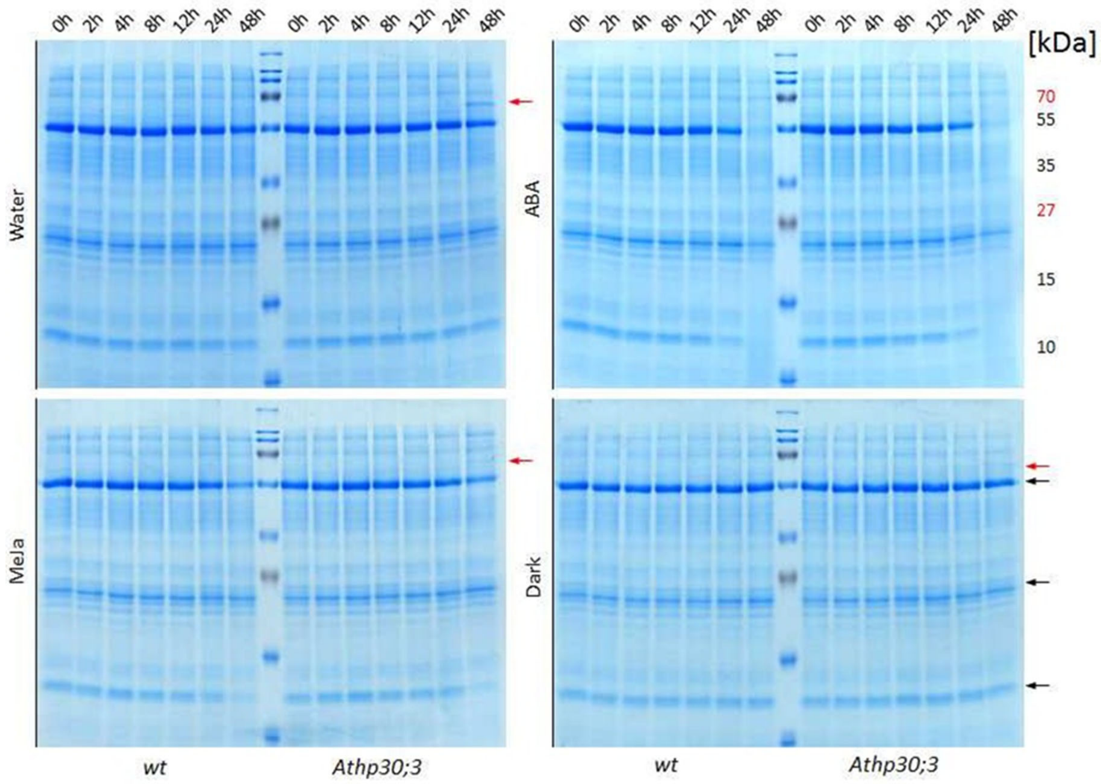

Figure S16. Protein accumulation patterns in A. thaliana wild-type and mutant Athp30;3 during senescence. Leaves were cut and incubated in 0.1 mM ABA, 45 µM MeJA or tap water or kept in continuous darkness for the indicated time periods. Coomassie staining of a representative separation of total proteins (20 µg/lane). Black arrows mark LSU (≈55 kDa), LHCII (≈27 kDa) and SSU (≈12 kDa), respectively, whereas the red arrow marks a protein that appears in the mutant but not in wild-type during senescence.

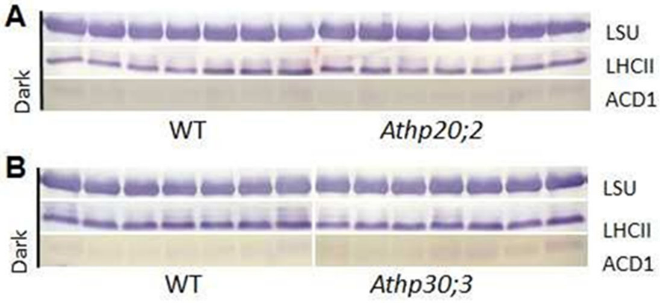

Figure S17. Chloroplast protein pattern of wild-type (WT) versus Athp20;2 and Athp30;3 mutant plants during dark-induced senescence. (**A**) Protein gel blot analysis of prominent chloroplast markers in 3 weeks-old wild-type (WT) versus Athp20;2 plants during dark-induced senescence ABA and MeJA treatments. **B**, as **A**, but depicting the results for wild-type (WT) versus Athp30;3 plants.

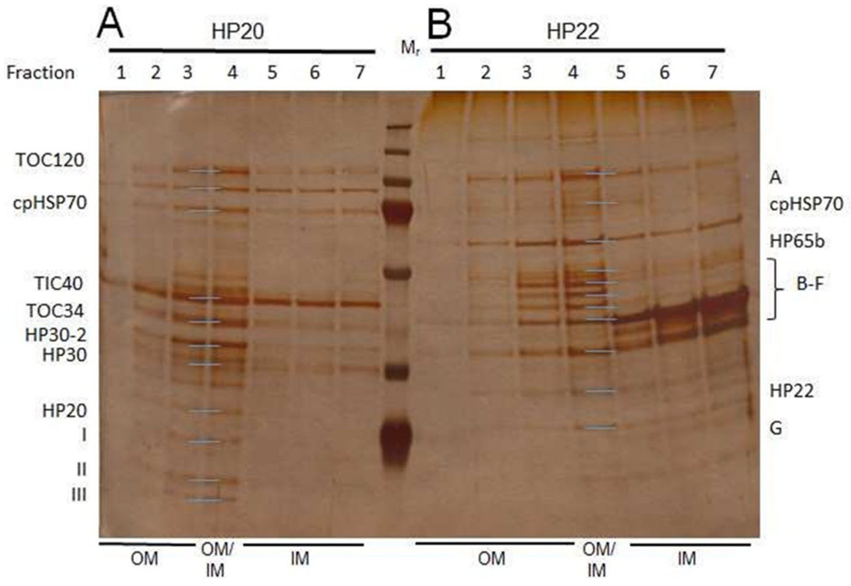

Figure S18. Identification of proteins interacting with HP20 (A) and HP22 (B) in chloroplasts, respectively. HP20-(His)6 and HP22-(His)6 were imported into chloroplasts from 4 weeks-old plants at 2.5 mM Mg⁻ ATP. Plastids then were ruptured hypotonically and crude envelopes separated by flotation on 20–38% sucrose gradients into outer envelope membranes (OM), inner envelope membranes (IM) and OM-IM junction complexes (OM-IM) formed by HP20-(His)6 and HP22-(His)6, respectively. Individual gradient fractions harvested and protein found in each fraction was resolved by SDS-PAGE and detected by silver staining. Replicate gels were run in parallel and used for Coomassie staining and protein sequencing. Positions of molecular mass standard (Mr) are indicated. (**A**) Pattern of plastid envelope proteins interacting with HP20-(His)6. Note the co-purification of HP20- (His)6 with HP30 and HP30-2 as well as other proteins forming the translocase for transit sequence- less proteins (cf. Figure 1). Some unidentified low molecular mass bands were designated I-III. (**B**) Pattern of plastid envelope proteins interacting with HP22-(His)6. Note the co-purification of HP22- (His)6 with HP65b but not HP30-2 in the OM-IM fraction. **A**–**G** define protein bands not discussed here.

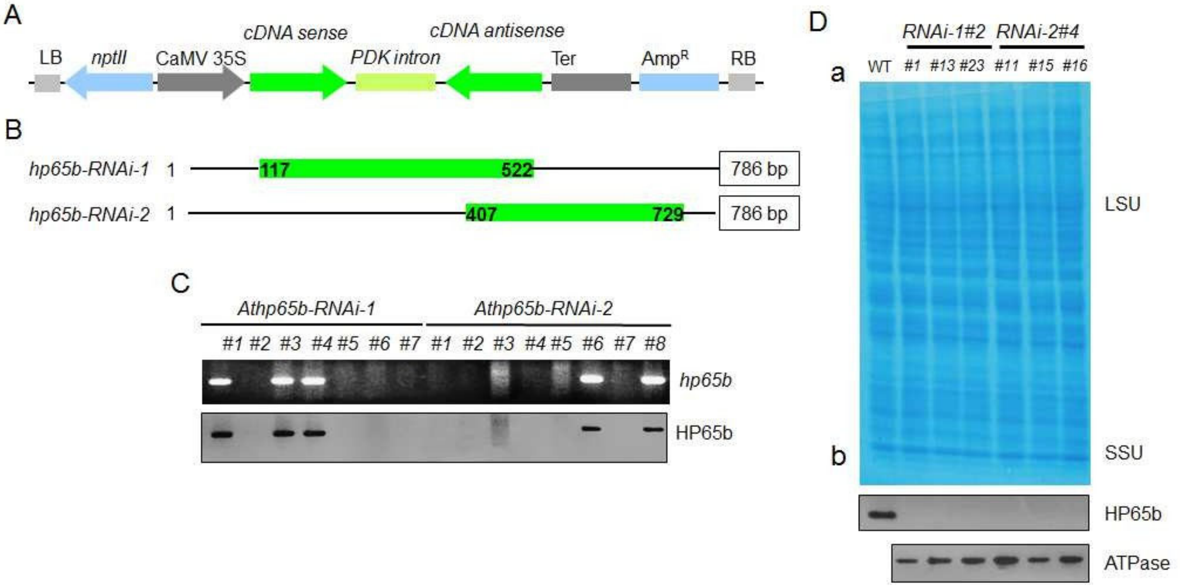

Figure S19. Identification and characterization of RNAi plants lacking HP65b. (**A**) Representation of RNAi constructs generated in the binary vector pArt27 containing the RNAi inducing-relevant components between left (LB) and right border (RB). (**B**) Representation of the mRNA sequences of HP65b that were selected, in sense direction. Abbreviations: nptII, kanamycin resistance gene (plant selection marker); CaMV 35S, 35S cauliflower mosaic virus promotor; PDK,

pyruvate orthophosphate dikinase; Ter, Terminator; AmpR, ampicillin resistance gene for bacterial selection. (**C**) Comparative Reverse transcription (RT)-PCR (upper panel) and Western blot (lower panel) analysis to characterize RNAi plants devoid of hp65b transcript and HP65b protein. (**D**) Accumulation of total leaf proteins (**a**) and HP65b as well as the β-subunit of the CF0-CF1 ATPase (ATPase) (**b**) in the offspring of Athp65bRNAi-1#2 and Athp65bRNAi-2#4 plants. LSU and SU define the large subunits and small subunits of ribulose-1,5-bisphosphate carboxylase/oxygenase. Shown are the results of Coomassie staining (**a**) and Western blotting (**b**) with specific antisera.

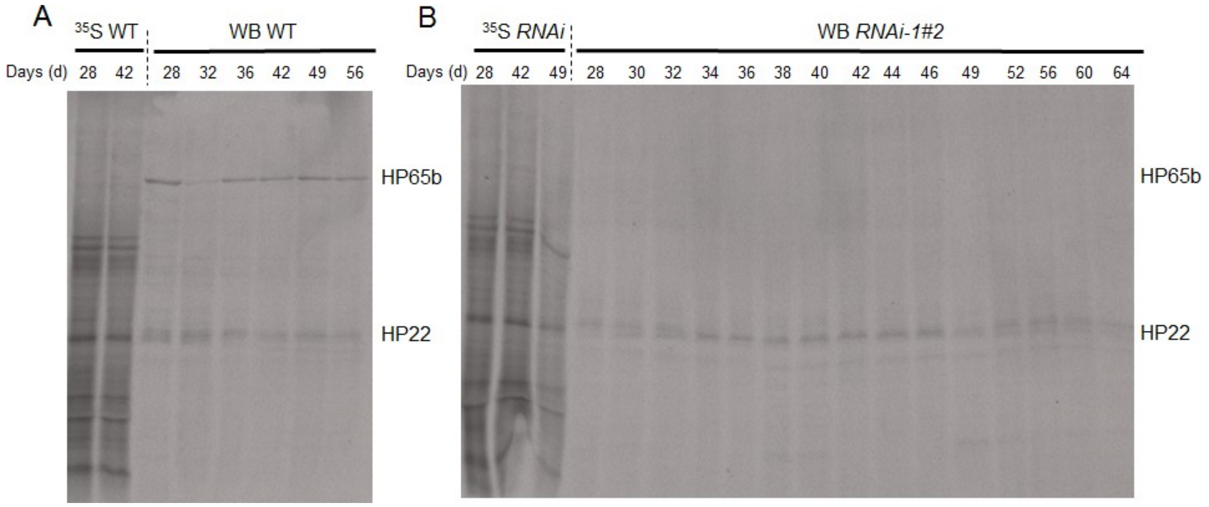

Figure S20. Analysis of HP65b and HP22 expression over the course of plant development. (**A**) Western blot (WB) analysis of HP65b and HP22 in 4-weeks-to-7-weeks-old Arabidopsis wild-type (WT) plants grown in 16h light/8 h dark-cycles at 60 μE m^{-2} s^{-1}. For comparison, the pattern of total leaf proteins that had been labelled with 35S-methionine for 1 h prior to leaf harvest are included (35S). **B**, as **A**, but showing the lack of HP65b protein in the generated hp65b-1#2 line. Note that the level of HP22 remains unaffected by the RNAi approach, highlighting the specificity of the assay.

2.2. Supplementary Tables

Table 1. Identification of plastid envelope proteins interacting with ceQORH-GFP.

Protein<br>PRAT Family Members	Gene	Mr	pI	Function	Reference
AtHP20	At4g26670	21.820	7.83	Translocation Channel (OM)	Ferro et al. [1]
AtHP30	At3g49560	27.982	10.21	Translocation Channel (IM)	Ferro et al. [1]
AtHP30-2	At5g24650	27.772	10.25	Translocation Channel (IM)	Murcha et al. [2]
TOC receptors and GTPases					
AtTOC120	At3g16620	119.92	4.73	Receptor	Bauer et al. [3]
AtTOC90	At5g20300	89.328	8.24	Receptor	Bauer et al. [3]
AtTOC34	At5g05000	34.076	10	GTPase	Jarvis et al. [4]
Chaperones and partners					
AtHSP93-V	At5g50920	103.41	6.73	ATP-driven import motor	Akita et al. [5]; Nielsen et al. [6] Chou et al. [7]
AtcpHSC70	At4g24280	76.507	4.81	ATP-driven import motor	Su and Li [8,9]
AtTIC40	At5g16620	48.902	5.07	Docking protein for HSP93	Stahl et al. [10]; Bedard et al. [11]
Redox components					
AtTIC55	At2G24820	60.607	8.87	Redox sensor	Caliebe et al. [12]

1. Ferro M, Salvi D, Brugière S, Miras S, Kowalski S, Louwagie M, Garin J, Joyard J, Rolland N (2003) Proteomics of the chloroplast envelope membranes from *Arabidopsis thaliana*. *Mol Cell Proteomics* **25**, 325-345.
2. Murcha MW, Elhafez D, Lister R, Tonti-Filippini J, Baumgartner M, Philippar K, Carrie C, Mokranjac D, Soll J, Whelan J (2007) Characterization of the preprotein and amino acid transporter gene family in *Arabidopsis*. *Plant Physiol* **143**, 199- 212.
3. Bauer J, Chen K, Hiltbunner A, Wehrli E, Eugster M, Schnell D, Kessler F (2000) The major protein import receptor of plastids is essential for chloroplast biogenesis. *Nature* **403**, 203-207.
4. Jarvis P, Chen L-J, Li H-m, Peto CA, Fankhauser C, Chory J (1998) An *Arabidopsis* mutant defective in the plastid general protein import apparatus. *Science* **282**, 100-103.
5. Akita M, Nielsen E, Keegstra K (1997) Identification of protein transport complexes in the chloroplastic envelope membranes via chemical crosslinking. *J Cell Biol* **136**, 983-994.
6. Nielsen E, Akita M, Davila-Aponte J, Keegstra K (1997) Stable association of chloroplastic precursors with protein translocation complexes that contain protein from both envelope membranes and a stromal Hsp100 molecular chaperone. *EMBO J* **16**, 935-946.
7. Chou ML, Chu CC, Chen LJ, Akita M, Li HM (2006) Stimulation of transit-peptide release and ATP hydrolysis by a cochaperone during protein import into chloroplasts. *J Cell Biol* **175**, 893-900.
8. Su PH, Li HM (2008) *Arabidopsis* stromal 70-kD heat shock proteins are essential for plant development and important for thermotolerance of germinating seeds. *Plant Physiol* **146**, 1231-1241.
9. Su PH, Li HM (2010) Stromal Hsp70 is important for protein translocation into pea and *Arabidopsis* chloroplasts. *Plant Cell*
10. **22**, 1516-1531.
11. Stahl T, Glockmann C, Soll J, Heins L (1999) Tic40, a new "old" subunit of the chloroplast protein import translocon. *J Biol Chem* **274**, 37467-37472.
12. Bédard J, Kubis S, Bimanadham S, Jarvis P (2007) Functional similarity between the chloroplast translocon component, Tic40, and the human co-chaperone, Hsp70-interacting protein (Hip). *J Biol Chem* **282**, 21404-2114.
13. Caliebe A, Grimm R, Kaiser G, Lübeck J, Soll J, Heins L (1997) The chloroplastic protein import machinery contains a Rieske-type iron-sulfur cluster and a mononuclear iron-binding protein. *EMBO J* **16**, 7342-7350.

Table S2. Chlorophyll accumulation in Athp20 and Athp30 mutant seedlings during greening.

	**Chlorophyll Content (µg/mg)**				
**Time (h)**	**WT**	***Athp20;1***	***Athp20;2***	***Athp30;2***	***Athp30;3***
0	n.d.	n.d.	n.d.	n.d.	n.d.
2	0.002	0.002	0.002	0.002	0.003
4	0.004	0.003	0.002	0.004	0.005
6	0.006	0.004	0.004	0.006	0.007
8	0.008	0.006	0.005	0.009	0.008
12	0.012	0.007	0.008	0.011	0.013
24	0.024	0.022	0.024	0.022	0.024

Chlorophyll measurements were made according to Porra et al. [1]. Chlorophyll contents were calculated on an equal fresh weight basis and refer to mean values obtained in three replicate experiments.

1. Porra RJ, Thompson WA, Kriedemann PE (1989) Determination of accurate extinction coefficients and simultaneous equations for essaying chlorophylls *a* and *b* extracted with four different solvents: verification of the concentration of chlorophyll standards by atomic absorption spectroscopy. *Biochim Biophys Acta* **975**, 384-394.

Table S3. Uptake of 35S-methionine into isolated plastids during plant etiolation and greening. Plastid amino acid uptake assays were conducted in the presence of 35S-methionine for wild-type (WT), Athp20;2 and Athp30;2 seedligs that had been grown for 3 d in darkness (Dark) or for 3 d or 7 d in white light (WL), respectively. Incorporation rates are given as decays per minute (dpm). Each row represents an individual experiment. ^{35}S-Met uptake (decays per minute).

**Wild-type**			***Athp20;2***			***Athp30;3***		
**3d Darkness**	**3d WL**	**7 d WL**	**3d Darkness**	**3d WL**	**7 d WL**	**3dDarkness**	**3 d WL**	**7 d WL**
17945	28123	37579	18456	29564	38532	18884	28325	37437
17442	27873	38006	19006	27437	38669	17833	27338	39322
17777	27007	37761	18429	28666	37998	18528	29521	38621

Table S4. Amino acid uptake into isolated plastids during leaf senescence of wild-type versus Athp20;2 and Athp30;3 mutant plants. Wild-type (WT), Athp20;2 and Athp30;3 plants were cultivated in alternate 16 h light/8 h dark cycles for the indicated time periods (weeks) and their chloroplasts isolated on Percoll. Plastid amino acid uptake was measured with 35S-methionine. Rates of amino acid uptake are gives as decay per minute (dpm). Each row represents an individual experiment. ^{35}S-Met uptake into chloroplasts isolated from plants of different age (dpm).

**1 Week**			**2 Weeks**			**3 Weeks**			**4 Weeks**		
**WT**	***Athp20;2***	***Athp30;3***	**WT**	***Athp20;2***	***Athp30;3*** **WT**	**WT**	***Athp20;2***	***Athp30;3***	**WT**	***Athp20;2***	***Athp30;3***
37579	38532	37437	28664	27112	26990<br>20744	20744	20552	21449	12775	12447	12666
38006	38669	39332	26210	24664	26548<br>20672	20672	19885	20888	12889	12649	12621
37761	37998	38621	27690	29003	28854<br>20821	20821	21222	20241	12338	12321	12489
